# SPRY4 as a Potential Mediator of the Anti-Tumoral Role of Macrophages in Anaplastic Thyroid Cancer Cells

**DOI:** 10.3390/cancers15174387

**Published:** 2023-09-01

**Authors:** Ana Teresa Pinto, Marta Pojo, Ricardo Rodrigues, Diana Pacheco Sousa, Rune Matthiesen, Ana Sofia Carvalho, Hans C. Beck, Carolina Pires, Rodrigo Eduardo, Joana Simões Pereira, Valeriano Leite, Branca Maria Cavaco

**Affiliations:** 1 Unidade de Investigação em Patobiologia Molecular (UIPM), Instituto Português de Oncologia de Lisboa Francisco Gentil (IPOLFG), 1099-023 Lisboa, Portugal; marta.pojo@ligacontracancro.pt (M.P.); rmrodrigues@ipolisboa.min-saude.pt (R.R.); diana.sousa@nms.unl.pt (D.P.S.); cs.pires@campus.fct.unl.pt (C.P.); rodrigo.eduardo@research.fchampalimaud.org (R.E.); jpereira@ipolisboa.min-saude.pt (J.S.P.); vleite@ipolisboa.min-saude.pt (V.L.); bcavaco@ipolisboa.min-saude.pt (B.M.C.); 2 Instituto de Biomedicina (iBiMED), Universidade de Aveiro, 3810-193 Aveiro, Portugal; 3 NMS Research, NOVA Medical School, Faculdade de Ciências Médicas (NMS|FCM), Universidade Nova de Lisboa, 1169-056 Lisboa, Portugal; rune.matthiesen@nms.unl.pt (R.M.); ana.carvalho@nms.unl.pt (A.S.C.); 4 Centre for Clinical Proteomics, Department of Clinical Biochemistry, Odense University Hospital, DK-5000 Odense, Denmark; hcbeck@health.sdu.dk; 5 Serviço de Endocrinologia, IPOLFG, 1099-023 Lisboa, Portugal; 6 NOVA Medical School, Faculdade de Ciências Médicas (NMS|FCM), Universidade Nova de Lisboa, 1169-056 Lisboa, Portugal

**Keywords:** anaplastic thyroid cancer, macrophages, invasion, migration, *SPRY4*, anti-tumoral

## Abstract

**Simple Summary:**

Anaplastic thyroid cancer (ATC) displays a high density of tumor-associated macrophages, which modulate invasiveness and tumor growth. However, the molecular mechanisms underlying the communication between tumor cells and macrophages in ATC tumor mass are still poorly understood. Our study observed bidirectional communication mechanisms between macrophages and ATC cells, in which macrophages influenced cancer cell viability and invasive phenotype and cancer cells modulated macrophage polarization. We identified *SPRY4* as a crucial mediator in this interplay, being a candidate tumor suppressor gene in this context, which may be useful to disclose new processes related to ATC aggressiveness that can lead to future therapeutic options.

**Abstract:**

Anaplastic thyroid carcinoma (ATC) is the most lethal subtype of thyroid cancer, with high invasive and metastatic potential, not responding to conventional treatments. Its aggressiveness may be influenced by macrophages, which are abundant cells in the tumor microenvironment. To investigate the role of macrophages in ATC aggressiveness, indirect co-cultures were established between ATC cell lines and THP-1-derived macrophages. Macrophages significantly increased both the migration and invasion of T235 cells (*p* < 0.01; *p* < 0.01), contrasting with a decrease in C3948 (*p* < 0.001; *p* < 0.05), with mild effects in T238 migration (*p* < 0.01) and C643 invasion (*p* < 0.05). Flow cytometry showed upregulation of CD80 (pro-inflammatory, anti-tumoral) and downregulation of CD163 (anti-inflammatory, pro-tumoral) in macrophages from co-culture with T235 (*p* < 0.05) and C3948 (*p* < 0.05), respectively. Accordingly, we found an upregulation of secreted pro-inflammatory mediators (e.g., GM-CSF, IL-1α; *p* < 0.05) in C3948–macrophage co-cultures. Proteomic analysis showed the upregulation of SPRY4, an inhibitor of the MAPK pathway, in C3948 cells from co-culture. *SPRY4* silencing promoted cancer cell invasion, reverting the reduced invasion of C3948 caused by macrophages. Our findings support that macrophages play a role in ATC cell aggressiveness. *SPRY4* is a possible modulator of macrophage–ATC cell communication, with a tumor suppressor role relevant for therapeutic purposes.

## 1. Introduction

Anaplastic (undifferentiated) thyroid cancer (ATC) is a rare and highly aggressive subtype of thyroid cancer and one of the most lethal malignancies [1,2]. ATCs are characterized by a complex background of genetic mutations, normally involving genes related to the MAPK signaling pathway [*BRAF* p.V600E (40–70%), *RAS* (24–43%)], the PI3K/AKT pathway [*PIK3CA* (10–18%), *PTEN* (10–15%)], and the *TP53* (60–80%) and *TERT* promotor (65–75%) [3,4,5]. Other genes, like *EIF1AX* [6], *CDKN2A/2B*, and *CDK4/6* [7,8], also play a role in ATC development. Since ATCs lost thyroid features, they no longer respond to either radioactive iodine or hormone therapies, used for differentiated thyroid cancer [9]. Surgery (when possible), radiotherapy and/or chemotherapy, or tyrosine kinase inhibitors are the recommended treatments, with multimodal therapies presenting greater results [10]. Nevertheless, the treatment response is poor, with an overall survival (OS) of 3 to 5 months and a progression-free survival (PFS) of 4 months [11,12,13]. Despite intensive research to improve ATC treatment using molecular targets, namely, against tyrosine kinase pathway members and immune checkpoints, there is still a lack of truly effective therapies able to overcome low survival rates [11,14,15]. The recently FDA-approved combination of BRAF (Dabrafenib) and MEK1/2 (Trametinib) inhibitors, referred to as DT therapy, has introduced a new paradigm for the management of ATC. This combination therapy promoted an increase in OS and PFS in ATC patients harboring the *BRAF* p.V600E mutation in their tumors [16,17]. However, DT resistance in ATC may occur due to acquired mutations.

Therapy resistance is not only determined by cancer mutations but also by the interplay of tumor cells with the tumor microenvironment (TME), which comprises a variety of cells, like stromal cells, immune cells, and endothelial cells, among others [18,19]. In ATCs, about 50–70% of the tumor mass is composed by tumor-associated macrophages (TAMs) [1,20], which exhibit ramified cytoplasmic extensions, forming a very dense network intermingled with cancer cells and blood vessels [20]. This huge macrophage infiltration also occurs, but at a much lower degree, in poorly differentiated thyroid cancer (54%), another type of advanced thyroid cancer, and in well-differentiated thyroid cancer (WDTC) (27%) [21]. Macrophage infiltration correlates with poor prognosis [21], namely, extrathyroidal extension, cervical lymph node metastases, and distant metastases [22], further suggesting a role for macrophages in thyroid cancer progression. 

TAMs are a very heterogeneous group of cells, which exhibit a spectrum of phenotypes between classically activated pro-inflammatory macrophages (M1) and anti-inflammatory/immunosuppressive macrophages (M2) [19,23]. In ATCs, TAMs appear to be predominantly polarized toward the M2 phenotype [20,21], characterized by CD163 positive staining, expression of pro-tumoral mediators [24,25], and secretion of factors that increase cancer cell invasion and stemness [25,26]. Considering that ATCs are generally resistant to conventional therapies and that macrophages represent a highly abundant cell population, macrophage targeting may represent a useful strategy to modulate ATC progression and response to therapy, particularly in cases not eligible for targeted therapy [27]. Accordingly, the pharmacological depletion of TAMs in thyroid cancer mouse models impaired tumor progression [28].

To achieve a better understanding of the role of macrophages in ATC aggressiveness and identify the associated biological mechanisms, we performed indirect co-cultures of four ATC cell lines (T235, T238, C643, and C3948) with THP-1-derived macrophages. We sought to determine how bidirectional communication between macrophages and ATC cell lines could contribute to the development of a pro- or anti-tumorigenic microenvironment.

## 2. Materials and Methods

### 2.1. Cell Lines

Four human ATC cell lines were used in this work: two harboring *BRAF* p.V600E mutation (T235 and T238) and two with wild-type *BRAF* (C643 and C3948). T235/T238 and C643 were kindly supplied by Dr. Lúcia Roque [Instituto Português de Oncologia de Lisboa Francisco Gentil (IPOLFG)] and by Dr. Paula Soares (IPATIMUP), respectively. C3948 was a new established ATC cell line previously characterized by our group [29]. THP-1, a human monocytic cell line, was used to obtain the macrophages. The cells were cultured in RPMI-1640 medium with HEPES (N-2-hydroxyethylpiperazine-N-2-ethane sulfonic acid) (Gibco, Life Technologies, Paisley, UK) (T235, T238, C3948, and THP-1) or without HEPES (Lonza, Verviers, Belgium) (C643), supplemented with 10% (*v*/*v*) fetal bovine serum (FBS) (Merck Millipore, Burlington, MA, USA), 1% (*v*/*v*) penicillin-streptomycin/amphotericin B Mix (Pan Biotech, Aidenbach, Germany), and 1% (*v*/*v*) L-glutamine (Gibco, Life Technologies). All cells were cultured at 37 °C, in a humidified 5% CO_2_ incubator. Mycoplasma-free cultures were confirmed using the universal mycoplasma detection kit (ATCC^®^ 30-1012K™, Manassas, VA, USA). Cell line authentication was confirmed by genotyping 10 short tandem repeat loci and comparing with those previously reported [29,30].

### 2.2. Macrophages 

**Figure 1:**
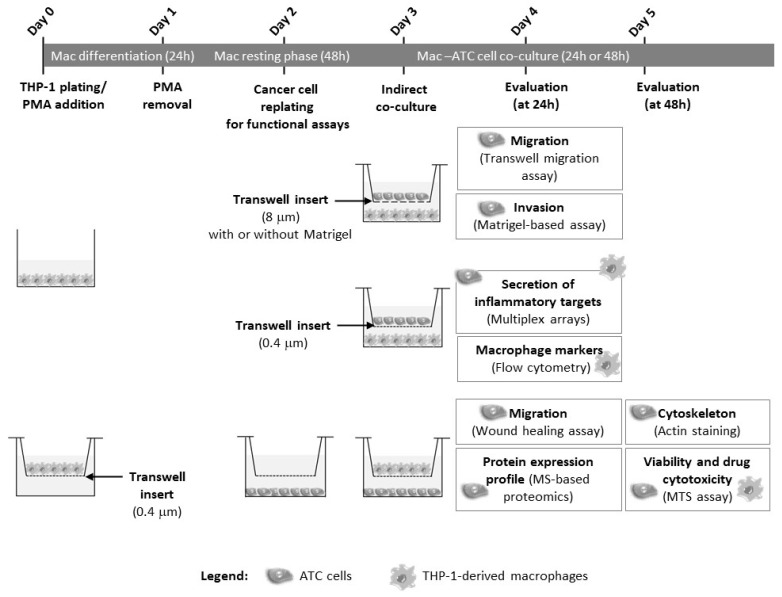
Schematic representation of the work. THP-1 monocytes were differentiated into macrophages for 24 h, in the presence of PMA. ATC cells (T235, T238, C3948, or C643) were seeded and stabilized for 24 h, after which both the ATC cells and the macrophages were indirect co-cultured for 24 or 48 h, using transwell inserts with different pore sizes (0.4 or 8 µm), coated or not with Matrigel, according to the purpose of each final assay. At 24 h after co-culture, the migration and invasion abilities of all four ATC cell lines, as well as the proteome profiles of T235 and C3948 cells, and the secretion of inflammatory targets from macrophage–cancer cell co-cultures were evaluated. The macrophage markers were also assessed. At 48 h after co-culture, cytoskeleton organization was evaluated. At this time point, the metabolic activity of ATC cells and macrophages, cultured in the presence or absence of cytotoxic drugs, was also evaluated. ATC, anaplastic thyroid cancer; Mac, macrophages; PMA, phorbol-12-myristate-13-acetate.

Macrophages were obtained from the monocytic THP-1 cell line, kindly provided by Dr. Raquel Gonçalves from i3S (Porto, Portugal). THP-1 cells were plated in different culture plate formats (24 well—2 × 10^5^ cells/well; 6 well—6–8 × 10^5^ cells/well; 100 mm plates—5.3 × 10^6^ cells/plate), according to the experimental purpose. Incubation with 200 nM of phorbol 12-myristate 13-acetate (PMA) for 24 h allowed monocyte differentiation into macrophages, which were maintained in the culture for an additional 48 h (resting phase) without PMA (Figure 1). PMA is widely used to induce monocyte differentiation into macrophages. 

### 2.3. Macrophage–ATC Co-Cultures

Macrophages and ATC cells (T235, T238, C643, and C3948) were plated separately in two independent compartments of a transwell system (Corning, Manassas, VA, USA): an upper compartment, consisting of a transwell insert with a microporous membrane, and a lower one. Cells were plated in transwell inserts of different porous size membranes or in the bottom compartment, depending on the assay, as detailed in this section. Transwell membranes with a smaller pore size (0.4 μm) allowed only the exchange of soluble factors, while those with larger pores (8 μm) allowed cell crossing, and for that reason the latter were used for the transwell-based invasion and migration assays. Both cell compartments were joined in an indirect co-culture, by transferring the upper compartment on the top of the other (Figure 1).

### 2.4. Transient Inhibition of SPRY4 Using siRNA-Mediated Silencing

*SPRY4* silencing in ATC cell lines (C3948 and T235) was performed using short interfering RNA (siRNA). Briefly, ATC cells were transfected using ON-TARGETplus Human SPRY4 siRNA-SMARTpool (Dharmacon, Lafayette, LA, USA) and the DharmaFECT^TM^ (Dharmacon, Lafayette, LA, USA) as a transfection reagent for 72 h, to achieve maximum silencing. The ON-TARGETplus Non-targeting pool siRNA (Dharmacon, Lafayette, LA, USA) was used as a transfection control. *SPRY4* silencing was evaluated using quantitative reverse transcriptase polymerase chain reaction (qRT-PCR), following the SYBR Green (Applied Biosystems, Waltham, MA, USA) protocol. For the data analysis, the relative gene expression was calculated by the ΔΔCt method [(ΔΔCt = (Ct target gene − Ct reference gene) treatment sample − (Ct target gene − Ct reference gene) control sample); Ct = threshold cycle]. Hypoxanthine phosphoribosyltransferase 1 (*HPRT1*) was used as the endogenous control gene. The fold change [FC = 2 − ΔΔCt] and the percentage of knockdown [%*KD* = (1 − FC) × 100] of the target gene in the treated cells were also calculated. 

### 2.5. Metabolic Cell Viability and Cytotoxic Drug Assay

The metabolic activity of ATC cells and macrophages, either from mono- or co-cultures (Figure 1), was evaluated in the presence or absence of cytotoxic drugs, using the MTS [3-(4,5-dimethylthiazol-2-yl)-5-(3-carbomethoxyphenyl)-2-(4-sulfophenyl)-2H-tetrazolium]-PMS (phenazine methyl sulfate) assay (Promega, Madison, WI, USA) according to the manufacturer’s instructions. The MTS assay was based on the reduction of MTS tetrazolium compound by metabolically active cells, generating a colored product. MTS reduction was considered a marker of cell viability, reflecting viable cell metabolism. About 24 h after ATC cell plating in 24-well plates (T235 and T238—2 × 10^4^ cells/well; C643 and C3948—1.25 × 10^4^ cells/well) in culture medium supplemented with 10% (*v*/*v*) FBS, the transwell inserts containing macrophages were transferred to the top of the cancer cell compartment. For cytotoxic drug assessment, monocultures or macrophage–cancer cell co-cultures were incubated with doxorubicin (2 mg/mL), paclitaxel (6 mg/mL), and cisplatin (1 mg/mL), kindly provided by Dr. Humberto Gonçalves from the Pharmacy of IPOLFG obtained, and diluted in cell culture medium at final concentrations of 0.5 µM, 0.5 µM, and 5 µM, respectively. After 48 h of incubation at 37 °C, the upper insert (with macrophages) and the respective bottom compartment (with cancer cells) were incubated separately with MTS. After 1 h, the absorbance was read, in duplicate, at 490 nm using the Microplate Absorbance Reader (iMARK^TM^; Bio-Rad Laboratories, Hercules, CA, USA). The metabolic viability was calculated relative to monoculture controls or untreated controls in the case of the cytotoxic test.

### 2.6. Migration and Invasion Assays

The migration ability of ATC cells was evaluated using transwell inserts with pore size membranes of 8 μm (Corning, Manassas, VA, USA). The inserts were pre-coated with the solubilized basement membrane preparation Matrigel™ (Corning, Manassas, VA, USA), which allowed the measurement of the ability of cells to attach, degrade the matrix, and migrate toward a chemoattractant (Figure 1). The ATC cells were plated, in duplicates, in 24-well transwell inserts (T238 and C643—4 × 10^4^ cells/insert; T235 and C3948—7.5 × 10^4^ cells/insert) with culture medium supplemented with 2% FBS (*v*/*v*). Immediately after plating, the inserts were transferred to the top of the compartment, where macrophages were cultured in medium supplemented with 10% FBS (*v*/*v*). The higher percentage of serum in the lower compartment acted as a chemoattractant for ATC cell migration and invasion. 

To evaluate the effect of *SPRY4* silencing on the macrophage-mediated invasion ability of ATC cells, siRNA silencing was performed in C3948 and T235 cells before co-culturing with macrophages. C3948 and T235 were seeded in 12-well Matrigel-based inserts (1.5 × 10^5^ cells/well), and *SPRY4* silencing was performed as described above. After 72 h of *SPRY4* silencing, the ATC cells were transferred to 24-well Matrigel-based invasion inserts (7.5 × 10^4^ cells/insert), in the presence or absence of THP-1-derived macrophages (2 × 10^5^ cells/well) on the bottom compartment. 

After 24 h, the cell culture medium was discarded, the porous membranes were washed with PBS and fixed with ice-cold methanol, and the non-migrating/non-invading cells were eliminated by scrubbing. Finally, the membranes were mounted in Vectashield^®^ Antifade Mounting Medium with 40,6-diamidino-2-phenylindole (DAPI) (Vector Laboratories, Burlingame, CA, USA), and the migrating/invading cells were visualized through an Olympus fluorescent microscope (CX30). Ten images per membrane, at 20× magnification, were acquired. The images were analyzed with Fiji software V1.53t, and the migrating/invasive cells were counted. ATC cells from co-culture with macrophages were compared with the monoculture. 

As an alternative approach to evaluate the migration ability of ATC cells, a wound-healing assay was also performed in some cell lines. Briefly, ATC cancer cells (C643 and C3948, 2 × 10^5^ cells/well) were plated in duplicate in six-well plates (Corning) (bottom compartment) for 24 h, after which a cell-free gap was created by scratching the confluent monolayer with a p200 pipette tip (time = 0 h). Then, the cell monolayer was washed with PBS, and the culture medium was replaced to remove cell debris. Subsequently, 0.4 μm transwell inserts (Corning) (top compartment), previously plated with THP-1-derived macrophages, were transferred into the culture system, allowing only the exchange of soluble factors between cancer cells and macrophages. The cells were co-cultured for 24 h. The wound was photographed at 0 h and at 24 h (Figure 1). The wound closure was measured using the Fiji software. 

### 2.7. Actin Filament Staining

To evaluate the cytoskeleton organization of the ATC cancer cells upon co-culture with macrophages, the actin filaments were stained using the phalloidin staining protocol. First, glass slides were covered with a solution of 0.2% (*w*/*v*) gelatin from porcine skin (G-1890, Sigma-Aldrich, St. Louis, MO, USA) in six-well plates (Corning, New York, NY, USA) and incubated at 37 °C for 15 min. After the removal of the gelatin solution, ATC cells (T238 and C643—1 × 10^4^ cells/well; T235—2 × 10^4^ cells/well; C3948—7 × 10^4^ cells/well) were plated on top of the gelatin-covered glass slides (Figure 1). Then, 0.4 μm transwell inserts, containing previously plated THP-1-derived macrophages, were transferred on the top of the cancer cells, allowing indirect co-culture. After 48 h, the culture medium was removed, and the ATC cells were washed twice with PBS and fixed with a solution of 4% (*w*/*v*) paraformaldehyde (PFA) (Sigma), for 20 min. Actin staining was performed with phalloidin-FITC (Sigma) (green) (diluted 1:100 in PBS 1×) for 20 min, in the dark, at room temperature. Glass slides were mounted with Vectashield mounting medium with DAPI (blue) (Vector Laboratories). Fluorescent images were acquired using a fluorescence microscope (Olympus CX30).

To investigate if *SPRY4* silencing promoted alterations in ATC’s morphology, ATC cells were plated in 12-well plates (1.5 × 10^5^ cells/well). After 72 h of *SPRY4* silencing, glass slides were added to the bottom wells of a 12-well plate and covered with a solution of 0.2% (*w*/*v*) gelatin and incubated at 37 °C for 15 min. Then, *SPRY4* silenced ATCs were plated (C3948—3 × 10^4^ cells/well; T235—8 × 10^4^ cells/well) on the gelatin-covered glass slides. THP-1 cells were plated in the 12-well plate’s inserts (1.7 × 10^5^ cells/insert). The actin filament phalloidin staining procedure was performed as described before.

### 2.8. Flow Cytometry 

The expression of typical macrophage markers, upon co-culture with cancer cells, was analyzed by flow cytometry. ATC cells were plated in six-well transwell inserts [T235 and C3948 cells (3 × 10^5^ cells/insert)] in culture medium supplemented with 2% (*v*/*v*) FBS for 24 h (Figure 1). Then, the inserts were transferred on the top of THP-1-derived macrophages, previously cultured in six-well plates. After 24 h of indirect co-culture, the macrophages were washed with PBS and incubated with a PBS solution containing 4 mM EDTA for 1 h at 37 °C. After detachment, the remaining adherent macrophages were scraped, the cells were collected and washed with PBS, and the pellet was resuspended in a 0.1% (*w*/*v*) bovine serum albumin (BSA) containing PBS solution. The macrophages were stained with anti-CD80 [conjugated to allophycocyanin (APC)] or anti-CD163 (APC) antibodies (Biolegend, San Diego, CA, USA). The cells were washed and fixed in a 2% PFA solution for at least 1 h and then stained with anti-CD68 (conjugated to FITC) antibody (Biolegend). Fifteen thousand events were acquired using a Becton Dickinson FACSCalibur flow cytometer (BD Biosciences, Franklin Lakes, NJ, USA). The acquired data were analyzed using the software FlowJo V10.7 (Tree Star Inc., Ashland, OR, USA).

### 2.9. Conditioned Medium Analysis

Indirect macrophage–T235 and macrophage–C3948 co-cultures were performed for 24 h, after which the conditioned medium of co-cultures and respective ATC monocultures was collected. Three independent co-culture experiments, with two experimental conditions (T235 and C3948 monocultures and macrophage co-cultures with T235 or C3948) each, were performed (Figure 1). To remove cells in suspension, the conditioned medium was centrifuged at 900× *g* for 5 min. The obtained supernatant was stored at −80 °C until further analysis. The analytes were measured by Eve Technologies (Calgary, AB, Canada) using the following multiplex assays: Cytokine/Chemokine 65-Plex Panel, Cytokine Array, TGF-beta 3-Plex, Angiogenesis Array & Growth Factor 17-plex Array, and MMP/TIMP Panel. The full list of analytes can be found in the Supplementary Methods. Analytes with most of the values outside of the detection range or equal to zero were excluded. 

### 2.10. MS-Based Proteomics 

The proteome profile of ATC cells (C3948 and T235), in monoculture and upon co-culture with macrophages, was evaluated through mass spectrometry (MS) analysis (Figure 1). ATC cells were plated in 100 mm dishes in culture medium supplemented with 2% FBS (*v*/*v*) (T235 and C3948—2 × 10^6^ cells/dish). After 24 h, THP-1-derived macrophages, previously cultured in transwell inserts, were transferred to the top of the cancer cells. After 24 h of indirect co-culture, the conditioned medium was collected for further analyte analysis, and cells were collected for proteomic analysis. For proteomic analysis, ATC cells were washed with PBS 1× and incubated with PBS 1× with 4 mM EDTA (pH 7.4), at 37 °C for 40 min or until cell detachment. The protein cell extract was accessed using a BCA protein assay kit (Pierce, Thermo Fisher Scientific, Waltham, MA, USA). Twenty micrograms of crude membrane fraction protein extract were digested using the protocol previously described [31,32]. The peptide samples were analyzed by nano-LC-MSMS (Dionex RSLCnano 3000) coupled to a Q-Exactive Orbitrap mass spectrometer (Thermo Scientific) in a similar way as previously described [33]. The obtained data from the 36 LC-MS runs were searched using VEMS V1.0 [34] and MaxQuant V1.6.2.1 [35]. The identified proteins were divided into evidence groups as defined by Matthiesen et al. [36]. The details are described in the Supplementary Methods.

### 2.11. Western Blot Validation

Western blot analysis was performed for SPRY4, in the two cell lines previously analyzed by proteomics. The 2D transwell co-cultures were performed, as described above, using C3948 and T235 ATC cell lines (2 × 10^5^ cells/well) and THP-1 cells (6 × 10^5^ cells/insert). An anti-SPRY4 rabbit polyclonal antibody (AB_2458875, Invitrogen, Waltham, MA, USA) was used. The secondary antibody incubation for SPRY4 was performed using goat polyclonal anti-rabbit conjugated with horseradish peroxidase (HRP) (#31460, Thermo Fisher Scientific, MA, USA). Alfa-tubulin was used as an endogenous control gene. The primary antibody incubation was performed using the anti-α-tubulin mouse monoclonal antibody (clone B-5-1-2; #T5168, Sigma-Aldrich, MO, USA), and a secondary incubation was performed using the goat polyclonal anti-mouse conjugated with HRP (#31430, Thermo Fisher Scientific, MA, USA). The analysis of SPRY4 protein expression by Western blot was performed in three independent assays.

To analyze the efficacy of *SPRY4* silencing at the protein level, the same procedure was performed, although using 10 μg of protein extracts and the SPRY4 rabbit monoclonal antibody (clone EPR12127, #ab176337, Abcam, Cambridge, UK). The secondary antibody incubation for SPRY4 was performed using goat polyclonal anti-rabbit conjugated with horseradish peroxidase (HRP) (#31460, Thermo Fisher Scientific, MA, USA). Beta-actin was used as an endogenous control gene. The primary antibody incubation was performed using the anti-β-actin mouse monoclonal antibody (clone AC-15, #A5441, Sigma-Aldrich, MO, USA), and the secondary antibody incubation was performed using the secondary antibody goat polyclonal anti-mouse conjugated with HRP (#31430, Thermo Fisher Scientific, MA, USA). The details are described in the Supplementary Methods.

### 2.12. Statistical Analysis 

The data from ATC or macrophages from co-culture (*n* = 4 for *SPRY4* silencing experiments, *n* = 3 for others) were compared with the respective monocultures. Most statistical analyses were performed using an unpaired parametric Student’s *t*-test. In the analysis of secreted inflammatory mediators, co-cultures were compared with monocultures using multiple paired Student’s *t*-tests, followed by a correction for multiple comparisons using the Holm–Šídák method. All statistical analyses were performed using Graph Pad prism (version 8.02 or 9.1.0 for secretome analysis). 

Regarding the proteomics data, the quantitative data from MaxQuant and VEMS were analyzed in R statistical programming language. The IBAQ values from the two programs were preprocessed by removing the common MS contaminants followed by log2(x + 1) transformation and quantile normalization, which were all subjected to statistical analysis utilizing the R package limma V3.48.3 [37], where the contrasts between co-culture and monoculture for the C3948 and T235 cell lines were specified. The correction for multiple testing was applied using the method of Benjamini and Hochberg [38].

All data are expressed as the mean (±standard deviation), and *p* values < 0.05 were considered statistically significant.

## 3. Results

To study the effect of macrophages on ATC cells, indirect co-cultures between macrophages and four ATC cell lines (T235, T238, C3948, or C643) were established, using transwell inserts with different pore sizes. We first characterized the effect of macrophages on cancer cells: (i) viability, in the presence or absence of cytotoxic drugs (MTS assay), (ii) cytoskeleton organization (actin staining), and (iii) migration (transwell/wound healing assays) and invasion (Matrigel-based assay) abilities, which are both hallmarks of malignancy, corresponding to the initial steps of the metastatic process. Then, to investigate the mechanisms associated with the observed functional differences, we analyzed (i) the typical macrophage markers by flow cytometry, (ii) the cancer cell protein expression profile through MS-based proteomics, and (iii) the secretion of inflammation-associated targets from the whole co-culture by multiplex arrays. 

### 3.1. Macrophages Decrease the Viability of T238 and C3948 ATC Cell Lines, While Maintaining the Intrinsic Chemoresistance Profile of Cancer Cells

**Figure 2:**
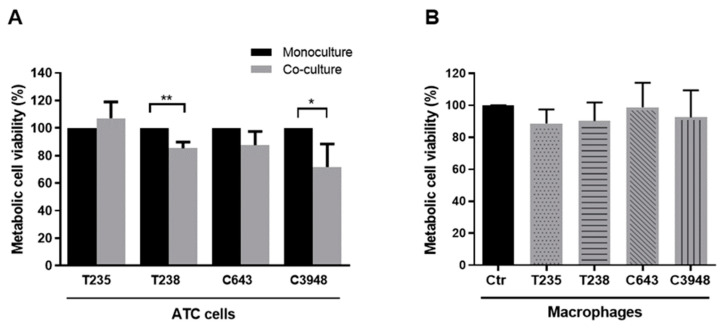
Viability of ATC cells and macrophages after indirect co-culture. MTS assay was used to evaluate the viability of (**A**) ATC cells (T235, T238, C3948, or C643) and (**B**) THP-1-derived macrophages upon indirect co-culture (*n* = 3). * *p* < 0.05, ** *p* < 0.01.

After indirect macrophage–cancer cell co-culture, the upper and lower compartments were separated, and the viability of macrophages and cancer cells was determined using the MTS assay. The results showed that the presence of macrophages significantly reduced the viability of T238 and C3948 ATC cells by about 15% (*p* < 0.01) and 30% (*p* < 0.05), respectively, while the viability of C643 and T235 cells was not significantly affected (Figure 2A). Regarding macrophages, their viability did not seem to be affected by co-culture with any of the ATC cells used (Figure 2B).

To evaluate if macrophages could confer drug resistance capacity to ATC cells, co-cultures, as well as cancer cell monocultures, were incubated with three drugs commonly used in the palliative care of ATC patients: cisplatin, paclitaxel, or doxorubicin. The viability, given by MTS assay, of drug-treated monocultures or co-cultures was compared with the respective untreated cultures (Figure S1). First, the viability pattern of all four ATC monocultures treated with the three drugs was quite similar, evidencing a strong reduction with paclitaxel and doxorubicin, with almost no alterations with cisplatin. Macrophages did not seem to alter this pattern, except when in co-culture with T238, where a significant increase (*p* < 0.05) in the viability of T238 cells upon exposure to doxorubicin was observed, when comparing with treated monoculture.

### 3.2. Macrophages Increase the Migration Ability of T235 ATC Cell Lines, While Decreasing That of T238 and C3948

**Figure 3:**
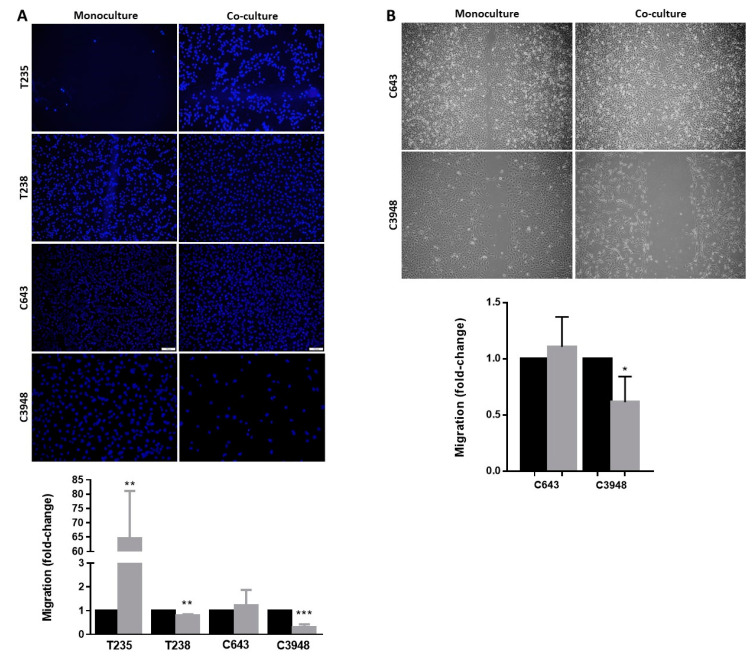
Migration ability of ATC cells upon co-culture with macrophages. (**A**) Migrating cells from transwells were stained with DAPI (blue) and counted, as presented in the graphs. (**B**) Wound size in wound healing-based migration assays was measured and presented in graphs. Co-cultures (gray) were compared with monocultures (black); (*n* = 3). * *p* < 0.05, ** *p* < 0.01, *** *p* < 0.001. Scale bar indicates 100 µm.

To evaluate how macrophages affected the migration ability of ATC cancer cells, two different assays were performed: transwell and wound healing-based migration assays. In the first one, cancer cells were plated on the top of 8 µm pore transwell inserts, which were transferred to the top of the macrophage culture. After 24 h, the migrating cells were photographed and counted, and the number of migrating cancer cells from the co-culture was compared with monoculture. The T235 cells significantly increased their migration ability in the presence of macrophages (*p* < 0.01), despite the great variability between independent experiments (Figure 3A). On the contrary, the migration of T238 cells slightly decreased (*p* < 0.01), and that of C3948 reduced more than 50% (*p* < 0.001), in the presence of macrophages. No alteration was observed to the intrinsic migration ability of C643 cells. Clearly, the T235 and C3948 cells lines were those with the strongest opposite effect upon co-culture with THP-1-derived macrophages (increase vs. decrease, respectively).

As an alternative method to validate promising data from the transwell-based migration assay, the conventional wound healing migration assay was also performed. After cancer cell plating, a scratch was performed to simulate a wound, and cells were indirectly co-cultured with macrophages for 24 h, after which the wound closure was evaluated. Due to the instability of a proper confluent monolayer in T235 and T238 cells, which is key for a correct wound scratch, this assay was only performed with C643 and C3948 cells. The results showed a decrease in the wound closure of the C3948 cells, corroborating the previous data regarding a reduced migration in the presence of macrophages (Figure 3B).

### 3.3. Macrophages Increase the Invasion Ability of T235 and C643 ATC Cell Lines, While Decreasing That of C3948

**Figure 4:**
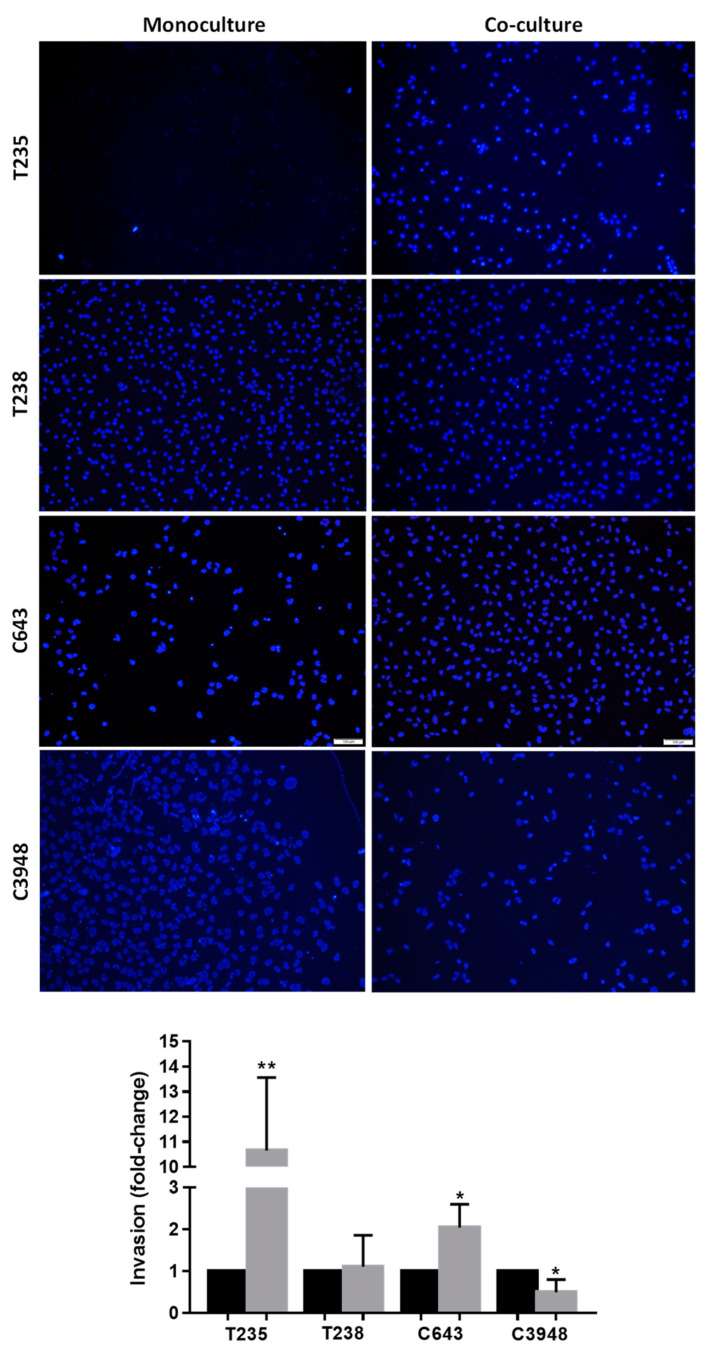
Invasion ability of ATC cells upon co-culture with macrophages. Invasive cells were stained with DAPI (blue) and counted, as presented in the graphs. Co-cultures (gray) were compared with monocultures (black); (*n* = 3). * *p* < 0.05, ** *p* < 0.01. Scale bar indicates 100 µm.

The invasion ability of ATC cancer cells was evaluated in monoculture or indirect co-culture with macrophages through Matrigel-based assays (Figure 4). Macrophages significantly promoted the invasion capacity of T235 (*p* < 0.01) and C643 (*p* < 0.05) cells, although a stronger effect was observed in T235, while they decreased the invasion of C3948 cells (*p* < 0.05) and maintained the intrinsic ability of T238. Again, the T235 and C3948 cell lines were those with the strongest opposite effect upon co-culture with THP-1-derived macrophages (increase vs. decrease, respectively).

### 3.4. Co-Culture with Macrophages Affects Differently ATC Cell Cytoskeleton

Considering cancer cell motility a key feature for migration and invasion capacities, we qualitatively evaluated motility-associated structures, such as lamellipodia, which are present in the mobile edges of the cells, and filopodia, which are thin cytoplasmic protrusions that extend from the leading edge of the mobile cells. Images from actin cytoskeleton staining (Figure S2) evidenced a different morphology of T235 and C643 cells upon co-culture with macrophages, contrasting with their round shape in monoculture. The effect seemed to be more prominent in T235 cells, which became elongated and acquired both filopodia and lamellipodia in the presence of macrophages, while C643 cells seemed already to present some lamellipodia when cultured alone in monoculture. These observations could support why increased migration and invasion in the presence of macrophages was statistically significant in T235 cells, but migration was not in C643. On the other hand, less evident differences were observed in the morphology and actin organization of T238 and C3948 cells in mono or co-culture with macrophages, suggesting that additional mechanisms may also account for the reduced migration and invasion above described, particularly for C3948.

### 3.5. Co-Culture of Macrophages with C3948 or T235 Exhibit a Different Cytokine Profile

**Figure 5:**
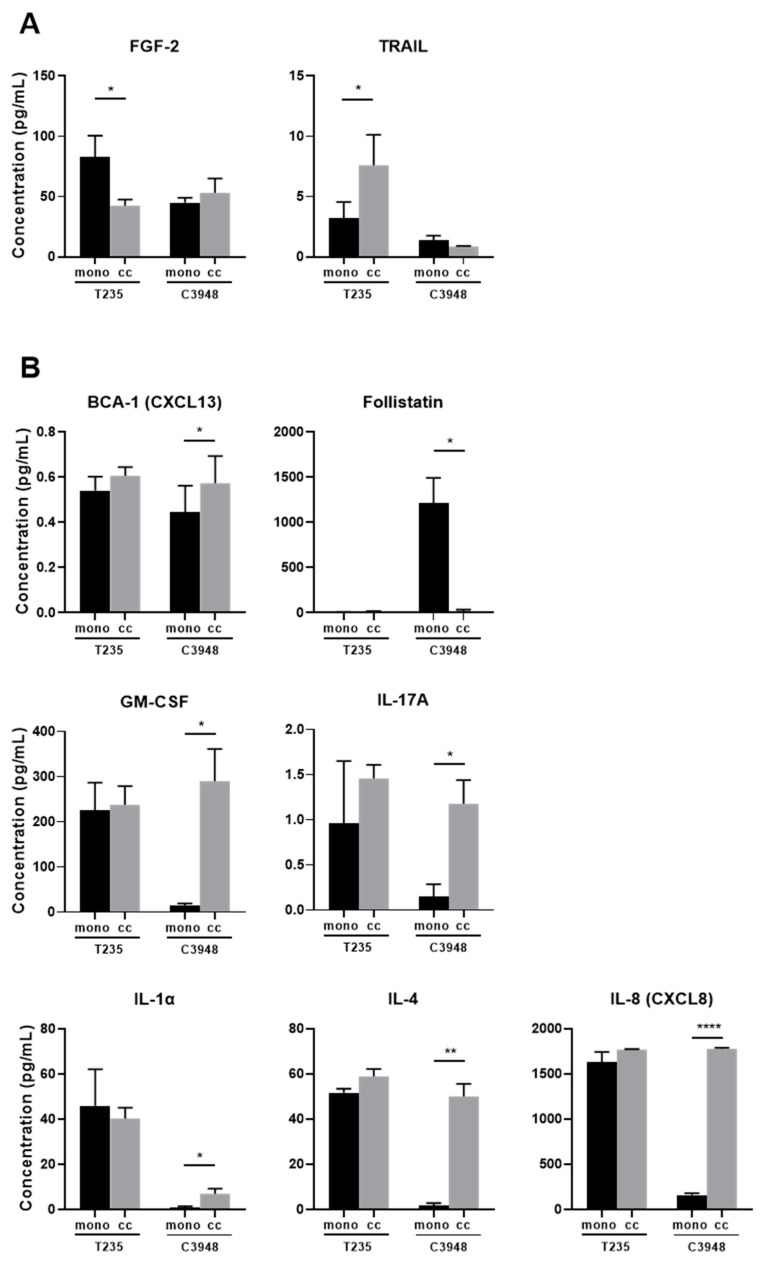
Targets differentially secreted in co-cultures between macrophages and T235 (**A**) or C3948 (**B**) ATC cells. The conditioned medium of T235/C3948-macrophage co-cultures and ATC monocultures was analyzed by multiplex arrays (*n* = 3). Co-cultures (cc, in grey) were compared with ATC monocultures (mono, in black). * *p* < 0.05, ** *p* < 0.01, **** *p* < 0.0001.

To investigate whether co-cultures of macrophages with T235 or C3948 had distinct inflammation-associated secretome profiles, which could help to explain the previous results (opposite effects), four panels of inflammatory cytokines, chemokines, and growth factors were evaluated in the conditioned medium of co-cultures and compared with ATC monocultures. From all targets analyzed (Supplementary Methods), 21 were excluded due to very low expression levels (zero or approximately zero), which could affect the data reliability. Only significant results in one or both cell line co-cultures are presented. We first looked at the common effects of macrophages when co-cultured with different ATC cell lines. We identified 16 targets significantly upregulated in both co-cultures when comparing with the respective ATC monocultures (Figure S3). For an additional 15 targets, the significance was only obtained for co-culture of macrophages with one of the cell lines, although a similar trend was found for the other one (Figure S4). We then focused on the specific effect of the presence of macrophages when co-cultured with each ATC cell line. Ideally, we were looking for a target significantly upregulated in one co-culture and downregulated in the other, or the opposite, which could help to justify the opposite effects in cancer cell migration and invasion observed in T235 (increase) and C3948 (decrease) upon co-culture with macrophages. We found that the FGF-2 levels significantly decreased in co-culture with T235 but not with C3948, while tumor necrosis factor-related apoptosis-inducing ligand (TRAIL) levels significantly increased (Figure 5A). On the other hand, the presence of macrophages significantly increased the secretion levels of BCA-1, GM-CSF, IL-17A, IL-1α, IL-4, and IL-8, while they significantly decreased follistatin levels in co-culture with C3948 but not with T235. IL-8 was the only target that passed the multiple testing correction (Figure 5B).

### 3.6. The Inflammatory Profile of Macrophages Is Different in Co-Culture with C3948 or T235 Cells

To investigate whether macrophages co-cultured with T235 were different from those co-cultured with C3948, THP-1-derived macrophages were collected, and the expression of CD80 and CD163 was evaluated by flow cytometry. 

**Figure 6:**
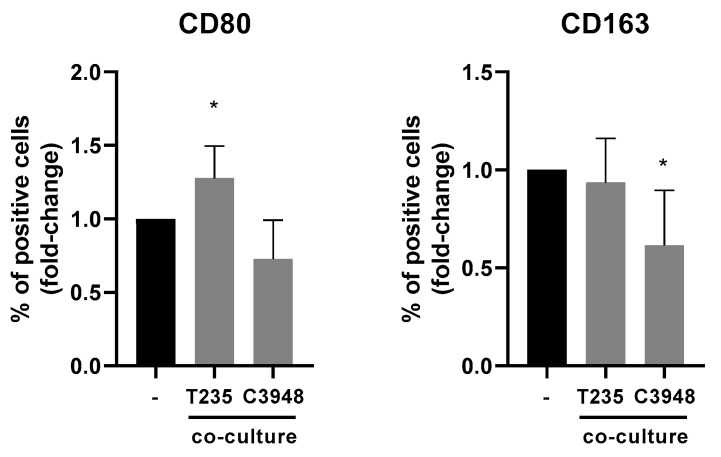
Characterization of CD80 and CD163 markers in macrophages co-cultured with ATC cells. Upon co-culture with T235 and C3948, THP-1-derived macrophages were collected, and the expression of CD80 and CD163 was evaluated by flow cytometry (*n* = 3). The fold change values were obtained after a comparison of macrophages from co-culture with C3948 or T235 with macrophage monoculture. * *p* < 0.05.

When comparing the expression of both markers in macrophages from co-culture versus monoculture, there was a significant increase (*p* < 0.05) of CD80 in macrophages from co-culture with T235 (with *BRAF* p.V600E mutation) and a significant decrease (*p* < 0.05) of CD163, in macrophages from co-culture with C3948 (without *BRAF* p.V600E mutation) cells (Figure 6).

### 3.7. Co-Culture with Macrophages Differentially Deregulates the Proteome Profile of C3948 and T235 Cells Unveiling SPRY4 as a Differentially Expressed Protein

**Figure 7:**
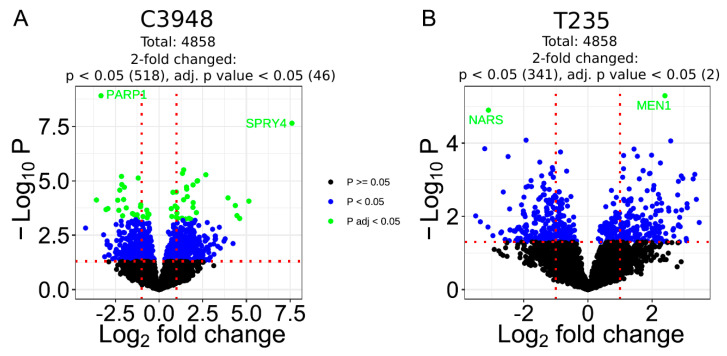
Targets deregulated in C3948 and T235 cancer cells after co-culture with macrophages. Volcano plots of the proteomic data of C3948 (**A**) and T235 (**B**) cells protein extracts. The proteome profile of ATC cells in co-culture was compared with monoculture. Differentially deregulated targets are represented in the x axis: downregulated ones are on the left side of the first dashed red line, while upregulated ones are on the right side of the second vertical line. The significance of the data is represented in the y axis. Significant targets are represented above the horizontal dashed red line, by blue (*p* < 0.05) and green (adj *p* value < 0.05) dots. Gene names of the most deregulated targets are indicated.

To investigate the signaling pathways underlying the opposite effect of macrophages on invasion and migration of the T235 (increase) and C3948 (decrease) cells, the proteome profile of these ATC cells, in co-culture with macrophages versus the respective monoculture, was analyzed through mass spectrometry. Overall, a total of 14,417 protein isoforms, collapsing into 4858 encoding genes, were identified. According to UniProt, of these, 1895 are annotated as membrane proteins (Figure S5), from which 115 are receptors. Co-culture with macrophages led to the significant deregulation of 57 proteins in C3948 cells but only two proteins in T235 cells (Figure 7; Tables S1 and S2).

The two proteins deregulated in T235 cells upon co-culture with macrophages were MENIN, a component of a histone methyltransferase complex encoded by the *MEN1* gene, and asparagine-tRNA ligase (AsnRS), encoded by the *NARS* gene, which were upregulated on average more than 5× and downregulated almost 10×, respectively. Regarding C3948, upon co-culture with macrophages, a functional analysis (Figure S6) showed there was a highly significant association with protein kinase D signaling (*p* < 0.001), given by its three members—polycystin-1 to 3 (PC1-3, encoded by PRKD1-3 genes), which were all upregulated. There was also an association with the sphingolipid biosynthetic process (*p* < 0.001), calcium-dependent protein kinase activity, the regulation of the nitric oxide biosynthetic process, and the response to interleukin-12 (*p* < 0.01). From the 57 targets significantly deregulated in C3948 by the presence of macrophages, 25 were downregulated and 32 upregulated; among these, 46 were, at least, two-fold deregulated. Among the most downregulated targets was poly [ADP-ribose] polymerase 1 (PARP1), while protein sprouty RTK signaling antagonist 4 (SPRY4) was undoubtedly the most upregulated target. Furthermore, enrichment analysis for all proteins identified significant altered pathways in both T235 and C3948 co-cultures (e.g., MYC targets and epithelial mesenchymal transition), as shown in the heat map representation (Figure S7).

**Figure 8:**
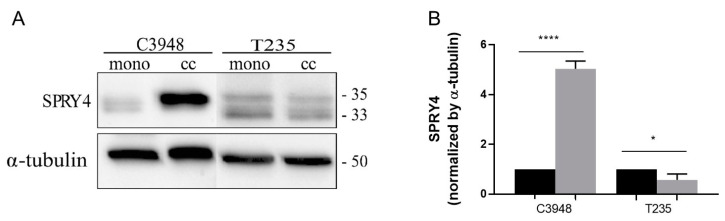
Validation of SPRY4 expression by Western blot. (**A**) Representative Western blot for SPRY4 (33 and 35 kDa) and α-tubulin (50 kDa) in macrophage-C3948/T235 co-cultures and respective ATC monocultures. The uncropped blots are shown in File S1. (**B**) SPRY4 Western blot protein quantification (*n* = 3) of co-cultures (gray), normalized by α-tubulin quantification and with the respective monoculture (black). * *p* < 0.05, **** *p* < 0.0001. mono, monoculture; cc, co-culture.

To further understand how macrophages can inhibit the migration and invasion of ATC cells, which is valuable information to develop new treatments against aggressive anaplastic thyroid tumors, we focused subsequent studies on C3948 cells, particularly on SPRY4. We validated the suggestive strong upregulation of SPRY4 by Western blot analysis in C3948 cells in co-culture with macrophages, when compared with the respective monoculture, using a different set of protein extracts than those used in proteomics (Figure 8). Interestingly, the proteomics data also indicated that T235 exhibited an approximately twofold decrease in SPRY4 expression upon co-culture with macrophages, although only moderately significant (*p* < 0.15), which was supported by Western blot analysis.

### 3.8. SPRY4 Silencing Reverts the Macrophage-Induced Reduction of C3948 Cell’s Invasion and Promotes Cytoskeleton Alterations in ATC Cells

**Figure 9:**
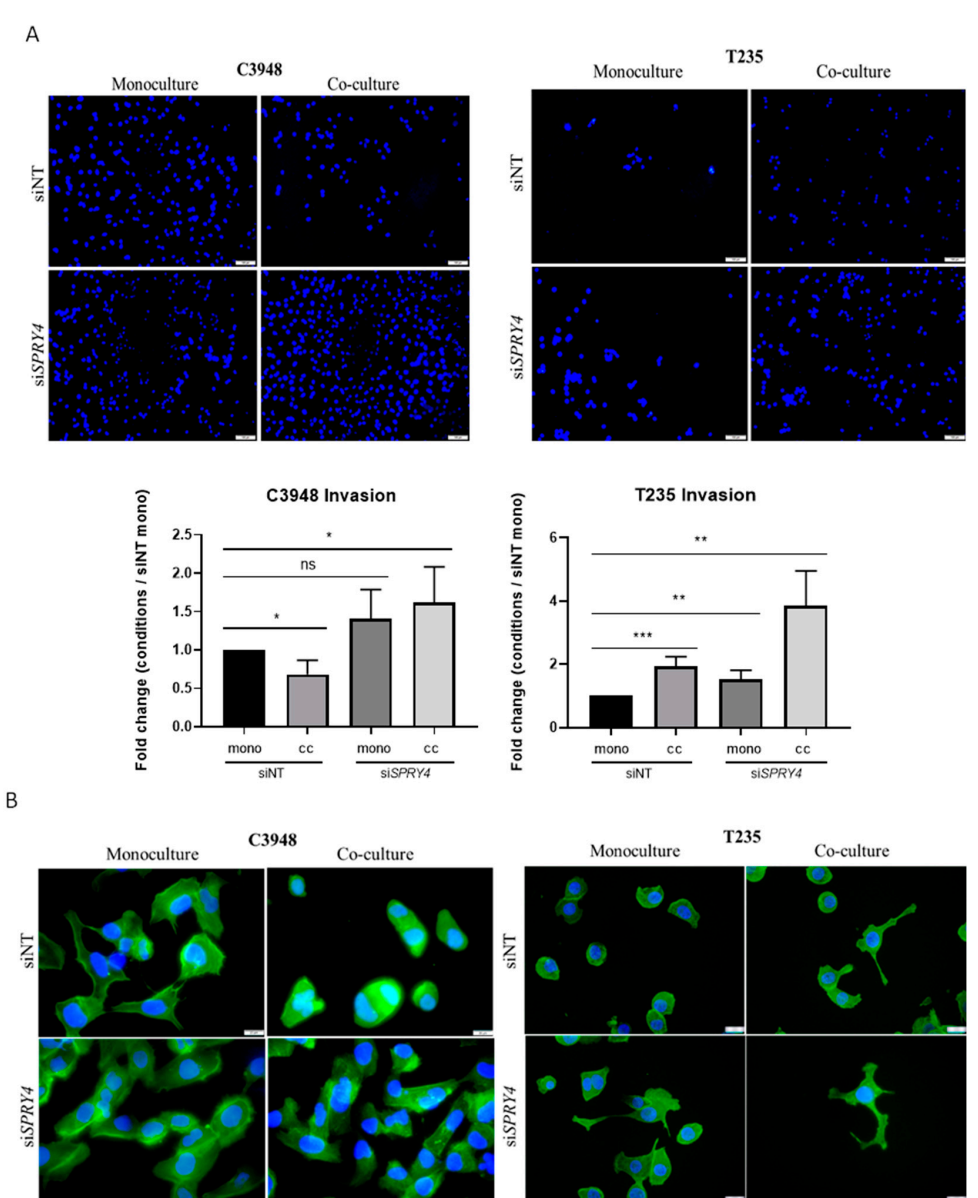
Effect of *SPRY4* silencing and THP-1-derived macrophages in ATC invasion and cytoskeleton alterations. *SPRY4* silencing increased C3948 and T235 invasion (**A**) in co-cultures (*n* = 4), which was confirmed by cytoskeletal alterations (*n* = 3) (**B**). DAPI (nuclei); Phalloidin (F-actin). (**A**) Image scales at 100 µm at 100× magnification, * *p* < 0.05, ** *p* < 0.01, *** *p* < 0.001, ns, non-significant. (**B**) Image scales at 20 µm at 400× magnification. si*SPRY4*—*SPRY4* silenced condition; siNT—non-target transfection control; mono—monoculture; cc—co-culture.

To evaluate the role of *SPRY4* in macrophage-mediated cancer cell invasion, transient *SPRY4* silencing, through siRNA, was performed in cancer cell monocultures and co-cultures (Figure S8). The efficient *SPRY4* silencing was demonstrated by a decrease in *SPRY4* expression by qRT-PCR and Western blot (Figure S9). Invasion assays were performed in the presence of *SPRY4* silencing (Figure 9A). The results demonstrated that using siRNA control (siNT), macrophages significantly altered invasion behavior in cancer cells, similar to that previously demonstrated with parental cells, that is, T235 cells increased (*p* < 0.001), while C3948 decreased (*p* < 0.05) their invasive potential, when compared with the respective monocultures. When *SPRY4* was inhibited (si*SPRY4*), in the absence of macrophages, invasion in both cell lines was increased, compared with siNT, although only significant in T235 (*p* < 0.01) cells. When *SPRY4* was inhibited, in the presence of macrophages, it also significantly promoted the invasion of both cancer cells, with C3948 restoring the invasion ability of siNT monoculture (*p* < 0.05), but at increased levels, and T235 exhibiting the highest level of invasion of all tested conditions. Complementarily, we also evaluated the actin cytoskeleton in monocultures and co-cultures, in the presence of siNT and si*SPRY4* (Figure 9B). The results are in accordance with the invasion data, showing that under *SPRY4* inhibition, macrophages promote an increase in cancer cell protrusions, suggesting higher cancer cell motility, which is essential for a successful invasion.

## 4. Discussion

ATC is one of the most aggressive tumors in humans, with a high invasive and metastatic potential. As ATC also exhibits a high density of TAMs, here we investigated whether macrophages could promote both migration and invasion, which together represent two essential first steps of the metastatic process. Surprisingly, we found that macrophages could have a dual role in ATC, depending on the cancer cell line they are co-cultured with. Our results showed that macrophages promoted T235 cancer cell migration and invasion, while they decreased these abilities in C3948 cells. Of note, we cannot exclude that the observed reduction in the viability of C3948 cells, upon co-culture with macrophages, may play a role in the decreased invasion and migration of these cancer cells after co-culture. The data from cytoskeleton analysis corroborated the migration and invasion data. In the presence of macrophages, T235 cells presented significant alterations in the actinic cytoskeleton, protrusions similar to filopodia and lamellipodia, which enable them to migrate and invade more than in monoculture, where these cells exhibited mostly a round-like shape. On the contrary, in the presence of macrophages, some C3948 cells acquired a round-like shape, although when in culture alone, they already exhibited lamellipodia structures, being quite different from T235 cells alone. 

The mechanisms underlying the macrophage-related dual phenotype, upon co-culture with T235 and C3948 cells, were further investigated. Importantly, the identification of targets associated with the observed effect in C3948 cells could help to understand how TAMs could be manipulated to reduce tumor aggressiveness. Therefore, we first focused on the secretion of inflammation-associated targets in ATCs alone versus in co-culture with macrophages. We hypothesized that a parallel increase or decrease in any factor in co-cultures of the two ATC cells with macrophages was most likely due to the presence of macrophages, rather than associated to the observed differences in migration and invasion. So, we particularly searched for factors that were differentially regulated in each co-culture, when compared with cancer cell monoculture. We found that in the T235–macrophage co-culture, TRAIL secreted levels were significantly higher compared with the T235 monoculture. Although TRAIL may play a dual role in tumorigenesis, in cancer cells resistant to TRAIL-mediated apoptosis induction, invasion and metastasis are activated [39]. 

On the other hand, the secretome of the C3948–macrophage co-cultures was significantly enriched in pro-inflammatory factors, like GM-CSF, IL1-α, IL-8, and IL-17A, when compared with C3948 monocultures, suggesting that these macrophages may be of the pro-inflammatory type. Pro-inflammatory macrophages can kill cancer cells directly, via mechanisms dependent on reactive oxygen species (ROS), reactive nitrogen species, and IL-1β and TNF-α production, or indirectly, through the activation of other immune cells, like cytotoxic T cells and NK cells [40]. This justifies the research interest in targeting TAMs into a pro-inflammatory phenotype, which in our results could be responsible for the reduced viability, migration, and invasion of the C3948 cells. Traditionally, GM-CSF has been used to *in vitro* generate pro-inflammatory dendritic cells/macrophages from mouse bone-marrow-derived monocytes [41], while LPS, IFN-γ, or a combination of both are used to polarize macrophages toward a pro-inflammatory profile [42]. Depending on the activator, pro-inflammatory macrophages may express high levels of IL-1B and IL-8 [42]. Additionally, both IL-1-α and IL-17A are strong pro-inflammatory cytokines with an immunosuppressive role. Unexpectedly, C3948-macrophage co-cultures also secreted high levels of IL-4, an interleukin used as an activator of a subgroup of anti-inflammatory (pro-tumoral) macrophages [42], which may suggest that macrophages from co-culture with C3948 cells may retain some anti-inflammatory features, exhibiting a mixed phenotype. All these molecules were barely secreted by C3948 cells alone and significantly increased in the presence of macrophages. Contrarily, follistatin secretion was completely abolished in the C3948–macrophage co-culture, when compared with the cancer cell monoculture. Since increased follistatin serum levels were found in thyroid cancer patients and correlated with advanced tumor aggressiveness [43], a strong reduction of follistatin secretion may support a role for macrophages in the reduced invasion and migration of C3948 cancer cells observed. 

Assuming that the C3948–macrophage co-cultures may be exhibiting a more pro-inflammatory phenotype than C3948 monocultures, it would be expected that macrophages from this co-culture would present a more pro-inflammatory phenotype (anti-tumoral) or, at least, a less anti-inflammatory (pro-tumoral) one. Accordingly, CD163 (anti-inflammatory) was significantly downregulated in macrophages from co-culture with C3948 cancer cells. On the other hand, a more anti-inflammatory phenotype (pro-tumoral) or, at least, a less pro-inflammatory (anti-tumoral) one would be expected for macrophages from co-culture with T235 cancer cells. However, CD80 (pro-inflammatory) was significantly upregulated in macrophages from co-culture with T235. This intriguing finding may be explained by the molecular characteristics of T235, mainly due to the presence of the *BRAF* p.V600E mutation. In thyroid cancer, Angell and colleagues demonstrated that there was a tendency for *BRAF* mutant tumors to have higher CD163 expression than CD80, but they also reported that this varied significantly between patients, with some having higher CD80 and others CD163 [44]. Although a broader panel of macrophage markers should have been used, rather than only evaluating the CD80 and CD163 markers, the obtained result can indeed make sense and help to explain the differences between the dual phenotypes of macrophages observed. 

To further explore the molecular mechanisms responsible for the dual phenotype of macrophages, a proteomic characterization was undertaken, and the obtained data suggested that SPRY4 was upregulated in C3948 cells when in co-culture with macrophages. A trend for decreased SPRY4 was also observed for T235 cells. This target was of particular interest, first due to its role in MAPK signaling, a crucial pathway in thyroid cancer, and in addition, our group had previously identified *SPRY4* as a novel candidate susceptibility gene for familial nonmedullary thyroid cancer (FNMTC) [45].

Sprouty proteins are feedback regulators of RTK, which restrain RTK-mediated signaling, thereby having critical roles in the regulation of cell proliferation, survival, and differentiation [46]. Particularly, *SPRY4* encodes for an inhibitor of the MAPK signaling pathway. It can be positioned upstream of RAS, where it impairs the formation of active GTP-RAS (RAS-dependent pathway) or downstream of RAS, by associating to the cysteine-rich domain (CRD) of RAF1 and blocking ERK activation (a RAS-independent pathway) [47]. *SPRY4* was also suggested to be a downstream target of the non-canonical WNT (Wingless-related integration site) signaling pathway, inhibiting cancer cell growth, migration, and invasion in non-small-cell lung cancer (NSCLC) [48]. Interestingly, a study by Hébrant and colleagues observed, through a microarray analysis, an increase in *SPRY4* mRNA expression in 10 out of 11 tumor samples from patients with ATC, when compared with papillary thyroid carcinomas (PTCs), which are less dedifferentiated tumors [49], supporting a role for this gene in ATC. 

**Figure 10:**
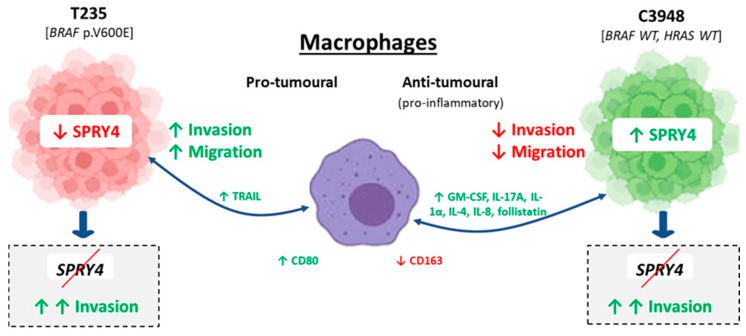
Bidirectional communication between THP-1-derived macrophages and ATC cell lines (T235 and C3948). Macrophages secrete specific extracellular mediators that promote an increase of invasion and migration of T235 cells, as well as alterations in the actinic cytoskeleton. On the other hand, secreted extracellular mediators from macrophages promote the opposite phenotype in C3948 cells. ATC cells secrete extracellular mediators that stimulate the expression of macrophage markers, such as CD80 and CD163. The proteomic profile of both cell lines changes in contact with macrophages, inducing the upregulation and downregulation of certain proteins, such as SPRY4, which was slightly downregulated in T235 and significantly upregulated in C3948. *SPRY4* knockdown induced cellular invasion in both cell lines, with or without contact with macrophages, and might be considered a possible candidate for a tumor suppressor gene, with an influence in the bidirectional communication between macrophages and ATC cells. ↑, increased expression or function; ↓, decreased expression or function.

To further explore the role of *SPRY4* in ATC cell–macrophage interaction, we silenced it in both T235 and C3948 cells, through an siRNA approach, and then assessed T235 and C3948 cells invasion ability, in monocultures and co-cultures. Interestingly, *SPRY4* inhibition reversed the macrophage-induced invasion reduction of C3948 cells previously observed. Somehow, it appears that the likely anti-tumoral effect of macrophages was abrogated by *SPRY4* silencing. In T235 cells, *SPRY4* inhibition promoted an increase in macrophage-mediated invasion, even higher than previously observed without *SPRY4* silencing. The data from the cytoskeleton analysis were consistent with the invasion data. In both contexts, *SPRY4* seems to be playing a tumor suppressor role (Figure 10). This tumor suppressor gene behavior of *SPRY4* has also been described by Tennis et al. in NSCLC [48]. However, in other cancers, namely, testicular germ cell tumors [50] and ovary cancers [51], *SPRY4* was suggested to behave as an oncogene. Previous functional *in vitro* studies developed by our group showed that siRNA-mediated *SPRY4* gene silencing induced a significant decrease in TPC-1 viable cells, suggesting that this gene may have an oncogenic activity in follicular-cell-derived thyroid cancer [45]. Comparing this study in well-differentiated thyroid cancer cells with the present results in ATC cells, it appears that *SPRY4* may have an opposite role in these subtypes of thyroid cancer. In the present study, we put forward that *SPRY4* could be a key mediator for the observed differences in the phenotypes of T235 (*BRAF* mutant) and C3948 (*BRAF* wild type) cell lines, under macrophage modulation in the tumor microenvironment. Although there could be an influence of *BRAF* mutations in *SPRY4* expression and effects, these cell lines present additional molecular alterations, as shown earlier by cytogenetic, transcriptome, and genome profiling (e.g., *TP53* mutation in C3948) [29,52], which may also take part in *SPRY4* mediator role of ATC cell–macrophage interaction.

It has been shown that ATC progression and therapeutic resistance may be greatly influenced by tumor-associated macrophages, which comprise the majority of the TME [1,20]. Only a subset of ATC cases are eligible for single-kinase targeted therapy [13]. Therefore, since the majority of ATCs are likely to be resistant to most therapeutic approaches, macrophage targeting may represent a useful strategy to modulate cancer progression and response to therapy [27,28]. Given that macrophages have dual roles in the TME, the elucidation of the molecular and cellular mechanisms involved in the macrophage–cancer cell interactions is crucial to define how to modulate this communication.

## 5. Conclusions

This study showed that macrophages seem to play a central role in modulating the aggressiveness (migration and invasion) of ATC cells, unveiling *SPRY4* as a possible mediator of this communication, with a tumor suppressor role. Reciprocally, ATC cells affect the expression of typical macrophage markers. 

Further studies on the role of *SPRY4* in ATC are required to expand the present knowledge of the mechanisms of action of macrophages in ATC progression and in the response to therapeutic approaches, which may also be useful in the development of future therapies.

## Data Availability

The mass spectrometry proteomics data that support the findings of this study have been deposited in ProteomeXchange Consortium [53] via the PRIDE [54] partner with the PXD044861 accession codes.

## Supplementary Materials

The following supporting information can be downloaded at https://www.mdpi.com/article/10.3390/cancers15174387/s1: Supplementary Methods: Evaluation of analytes in conditioned medium, MS-based proteomics and Western blot validation; Figure S1: Drug resistance of ATC cells in the presence of macrophages; Figure S2: Actin cytoskeleton organization of ATC cells upon co-culture with THP-1-derived macrophages; Figure S3: Secreted proteins significantly upregulated by the presence of macrophages in co-cultures with both ATC cell lines; Figure S4: Secreted protein targets significantly upregulated in at least one of the cell line co-cultures but with the same tendency in both; Figure S5: Venn diagram displaying overlap in identified proteins with UniProt annotated membrane proteins and receptors; Figure S6: Proteomic analysis of C3948 ATC cells after co-culture with macrophages; Figure S7: Heat map displaying −log10 P enrichment significance of cancer hallmark proteins encoded as colors when submitting regulated proteins for C3948 and T235 (co-culture vs. monoculture); Figure S8: Schematic representation of the experimental procedure after *SPRY4* silencing; Figure S9: Efficient *SPRY4* silencing in ATC cell lines; File S1: Full pictures of the Western blots. Tables S1 and S2: Proteomic data.
