# Supplementary material for: SPRY4 as a Potential Mediator of the Anti-Tumoral Role of Macrophages in Anaplastic Thyroid Cancer Cells
Source: Cancers (Basel). 2023 Sep 1;15(17):4387. doi: 10.3390/cancers15174387 (PMC10487195; doi:10.3390/cancers15174387)

Western blot gels

1. Western blots for SPRY4 protein validation (Figure 8)

SPRY4

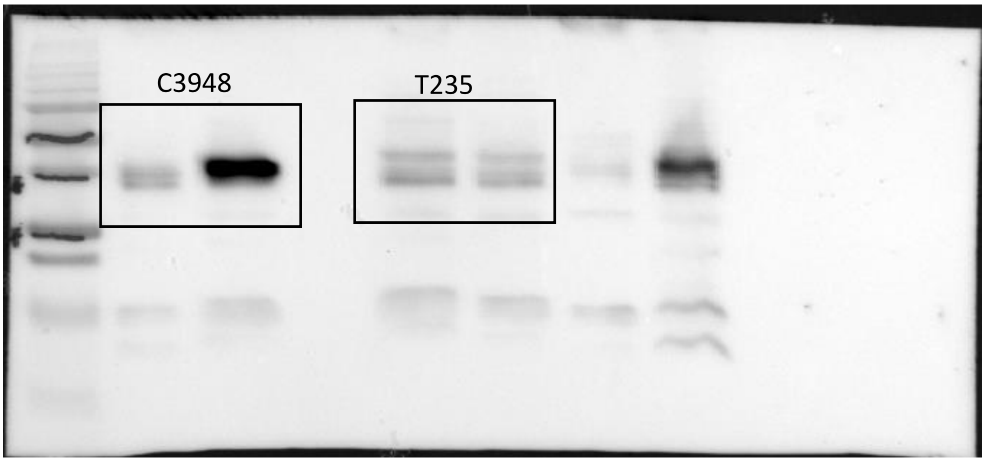

α-tubulin

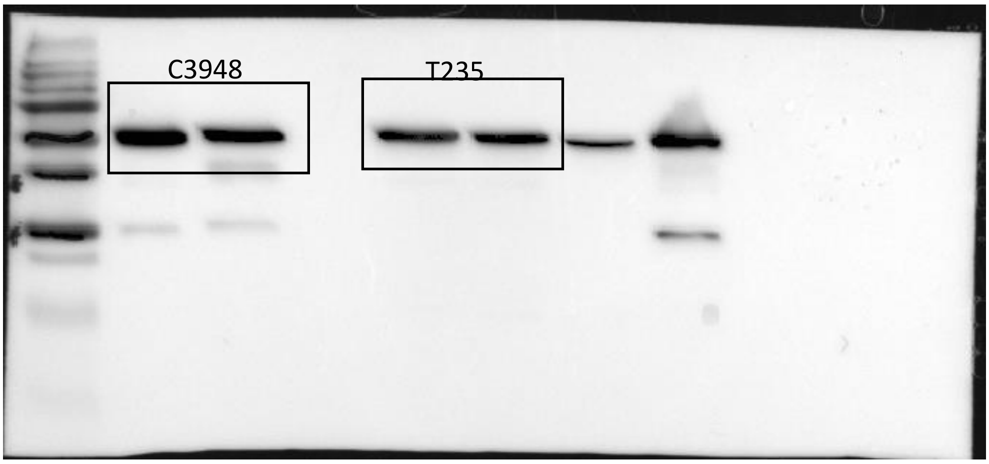

2. Western blots for SPRY4 silencing (Figure S9)

SPRY4 - T235

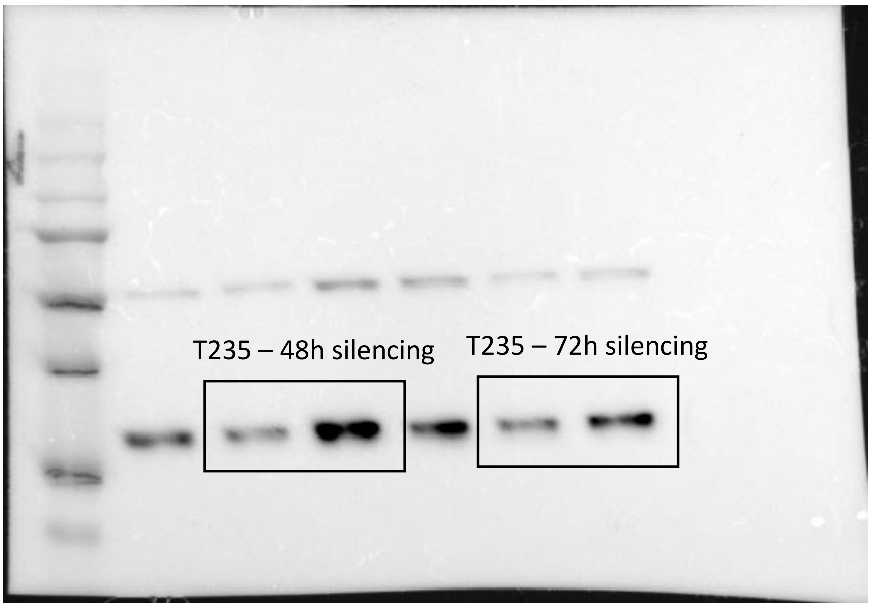

β-actin – T235

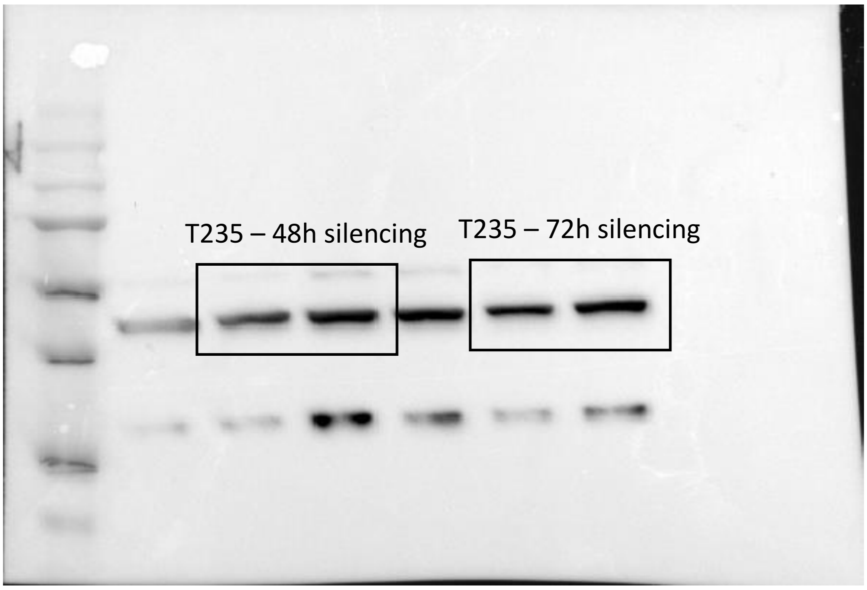

SPRY4 – C3948

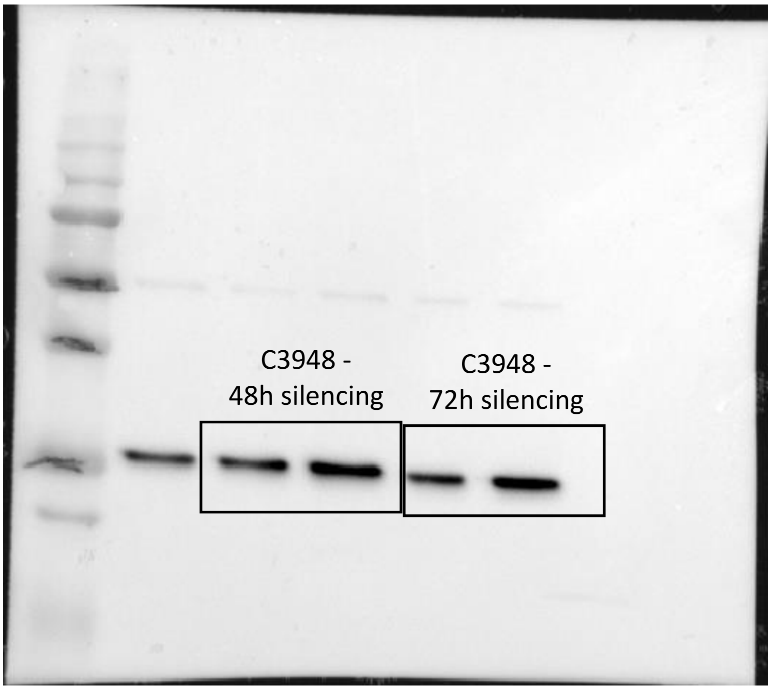

β-actin – C3948

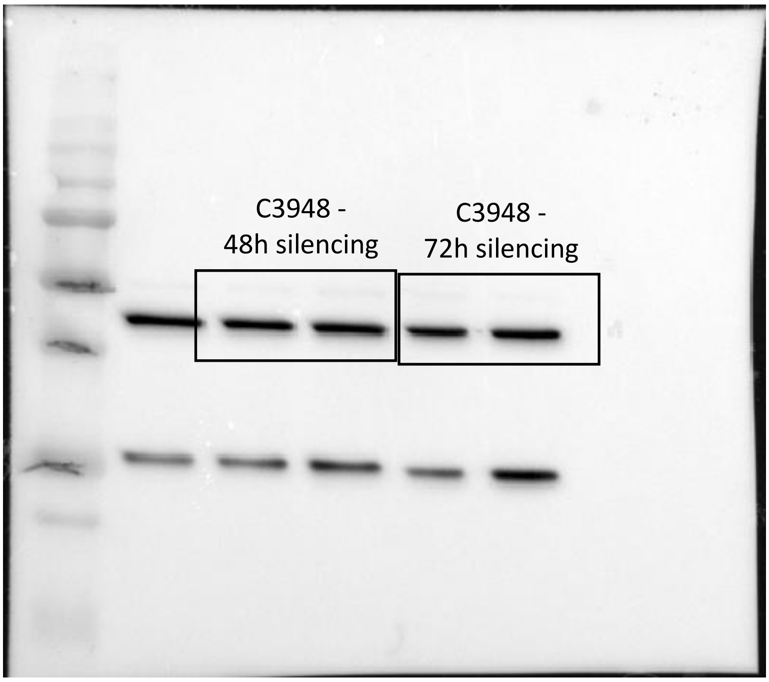

Supplement: Supplementary file 1 [file cancers-15-04387-s001.zip › cancers-2509168-File S1.pdf]
