# Supplementary material for: SPRY4 as a Potential Mediator of the Anti-Tumoral Role of Macrophages in Anaplastic Thyroid Cancer Cells
Source: Cancers (Basel). 2023 Sep 1;15(17):4387. doi: 10.3390/cancers15174387 (PMC10487195; doi:10.3390/cancers15174387)
Supplement: Supplementary file 1 [file cancers-15-04387-s001.zip › cancers-2509168-Methods and Figures.pdf]

SPRY4 as a potential mediator of the anti-tumoral role of macrophages in anaplastic thyroid cancer cells

Ana Teresa Pinto [1,2,*,†], Marta Pojo [1,†], Ricardo Rodrigues [1,†], Diana Pacheco Sousa [1], Rune Matthiesen [3], Ana Sofia Carvalho [3], Hans C. Beck [4], Carolina Pires [1], Rodrigo Eduardo [1], Joana Simões Pereira [1,5], Valeriano Leite [1,5,6] and Branca Maria Cavaco [1]

1 Unidade de Investigação em Patobiologia Molecular (UIPM), Instituto Português de Oncologia de Lisboa Francisco Gentil (IPOLFG), 1099-023 Lisboa, Portugal;
2 Instituto de Biomedicina (iBiMED), Universidade de Aveiro, 3810-193 Aveiro, Portugal
3 NMS Research, NOVA Medical School, Faculdade de Ciências Médicas (NMS|FCM), Universidade Nova de Lisboa, 1169-056 Lisboa, Portugal;
4 Centre for Clinical Proteomics, Department of Clinical Biochemistry, Odense University Hospital, DK-5000 Odense, Denmark;
5 Serviço de Endocrinologia, IPOLFG, 1099-023 Lisboa, Portugal
6 NOVA Medical School, Faculdade de Ciências Médicas (NMS|FCM), Universidade Nova de Lisboa, 1169-056 Lisboa, Portugal
* Correspondence: anapinto@ua.pt
† These authors contributed equally to this work.

Supplementary Methods

Evaluation of analytes in conditioned medium

Four panels commercially available at Eve Technologies were used for the evaluation of analytes in conditioned medium (Calgary, Canada). The full list of analytes included in the Cytokine/Chemokine 65-Plex Panel (HD65) included: secondary lymphoid-tissue chemokine (6CKine/CCL21); B cell-attracting chemokine 1 (BCA-1/CXCL13); cutaneous T-cell-attracting chemokine (CCL27/CTACK); epidermal growth factor (EGF); epithelial neutrophil-activating peptide-78 (ENA-78/CXCL5); eotaxin-1-3 (CCL11, CCL24, CCL26); fibroblast growth factor 2 (FGF-2); Fms-related tyrosine kinase 3 ligand (Flt-3L); fractalkine (CX3CL1); granulocyte colony-stimulating factor (G-CSF); granulocyte-macrophage colony-stimulating factor (GM-CSF); growth-regulated alpha protein (GROα/CXCL1); T lymphocyte-secreted protein I-309 (I-309/ CCL1); interferon alpha 2 (IFNα2) and gamma (IFNγ); IL-1α, IL-1β, IL-2, IL-3, IL-4, IL-5, IL-6, IL-7, IL-8 (CXCL8), IL-9, IL-10, IL-12 (p40), IL-12 (p70), IL-13, IL-15, IL-16, IL-17α, IL-18, IL-20, IL-21, IL-23, IL-28α, IL-33 and IL-1 receptor antagonist (IL-1RA); IFNγ-induced protein 10 (IP-10/CXCL10); leukemia inhibitory factor (LIF); monocyte chemoattractant protein 1-4 (MCP-1/CCL2; MCP-2/CCL8; MCP-3/CCL7; MCP4/CCL13), macrophage-derived chemokine (MDC/CCL22); macrophage inflammatory protein 1-alpha (MIP-1α/CCL3), 1-beta (MIP-1β/CCL4) and 1-delta (MIP-1δ/CC15); platelet derived growth factor (PDGF)-AA and PDGF-AB/BB; regulated upon activation, normal T cell expressed and secreted (RANTES/CCL5); soluble CD40 ligand (sCD40L); stem cell factor (SCF); stromal cell-derived factor 1-alpha+beta (SDF-1αβ/CXCL12); thymus and activation-regulated chemokine (TARC/CCL17); transforming growth factor alpha (TGFα); tumour necrosis factor alpha (TNFα) and beta (TNFβ); thyroid peroxidase (TPO); tumor necrosis factor ligand superfamily member 10 (TRAIL); thymic stromal lymphopoietin (TSLP); and vascular endothelial growth factor A (VEGFA).

The Cytokine Array, TGF-beta 3-Plex (TGFB1-3) included: transforming growth factor 1-3 (TGFβ1, TGFβ2, TGFβ2). The full list of analytes included in the Angiogenesis Array & Growth Factor 17-plex Array (HDAGP17) included: angiopoietin-2 (ANG-2); endoglin (ENG); endothelin-1 (ET-1); epidermal growth factor (EGF); fibroblast growth factor 1 (FGF-1) and 2 (FGF-2); follistatin (FS); granulocyte colony-stimulating factor (G-CSF); growth/differentiation factor 2 (GFF-2/BPM-9); proheparin-binding EGF-like growth factor (HB-EGF); hepatocyte growth factor (HGF); IL-8 (CXCL8); leptin; placenta growth factor (PIGF); vascular endothelial growth factor A (VEGFA), (VEGFC) and D (VEGFD).

The matrix metalloproteinases (MMP)/ tissue inhibitor of metalloproteinases (TIMP) Panel (HMMP/TIMP-C,O) included: interstitial collagenase 1 (MMP-1); gelatinase A (MMP-2); stromelysin-1 (MMP-3); matrilysin (MMP-7); neutrophil collagenase (MMP-8); gelatinase B (MMP-9); stromelysin-2 (MMP-10); macrophage metalloelastase (MMP-12); collagenase-3 (MMP-13); metalloproteinase inhibitor 1 (TIMP-1), 2 (TIMP-2), 3 (TIMP-3) and 4 (TIMP-4).

MS-based proteomics

The proteome profile of ATC cells (C3948 and T235), in monoculture and upon co-culture with macrophages, was evaluated through mass spectrometry (MS) analysis. ATC cells were plated in 100 mm dishes in culture medium supplemented with 2% FBS (v/v) (T235 and C3948 - $2x10^6$ cells/dish). After 24 h, THP-1 derived macrophages, previously cultured in transwell inserts, were transferred to the top of cancer cells. After 24 h of indirect co-culture, the conditioned medium was collected for further analyte analysis and cells were collected for proteomic analysis. For proteomic analysis, ATC cells were washed with PBS 1x and incubated with PBS 1x with 4 mM EDTA (pH 7.4), at 37 °C for 40 min or until cell detachment. Cells were collected, washed 3 times with cold PBS and centrifuged at 700x *g* for 5 min. Cell pellets were stored at -80 °C for further proteomic analysis. Cell pellet was homogenized with ice-cold Cell Homogenization Media (CHM) (10 mM HEPES-NaOH pH 7.5, 1 mM EGTA, 25 mM KCl) and complete Protease Inhibitor Cocktail tablets (Roche) with a Sonifer SFX 150-microtip (Branson Digital). For plasma membrane preparation, CHM containing 1 M sucrose was added to a final concentration of 0.25 M and centrifuged at 3 000x *g* for 15 min, 4 °C. The supernatant was sequentially centrifuged for 1 h at 100,000x *g*, 4 °C in an ultracentrifuge L100XP (Beckman Coulter). The pellet corresponded to a crude membrane fraction. Protein cell extract was accessed by BCA protein assay kit (Pierce, Thermo Fisher Scientific). Twenty micrograms of crude membrane fraction protein extract were digested using the protocol previously described (1).

Protein solution containing SDS and DTT were loaded onto filtering columns and washed exhaustively with 8 M urea in HEPES buffer (2). Proteins were reduced with DTT and alkylated with IAA. Protein digestion was performed by overnight digestion with trypsin sequencing grade (Promega).

Peptide samples were analyzed by nano-LC-MSMS (Dionex RSLCnano 3000) coupled to a Q-Exactive Orbitrap mass spectrometer (Thermo Scientific) in a similar way as previously described (3). Briefly, the samples (5 µL) were loaded onto a custom-made fused capillary pre-column (2 cm length, 360 µm OD, 75 µm ID) with a flow of 5 µL per min for 7 min. Trapped peptides were separated on a custom-made fused capillary column (20 cm length, 360 µm outer diameter, 75 µm inner diameter) packed with ReproSil Pur C18 3-µm resin (Dr. Maish, Ammerbuch-Entringen, Germany) with a flow of 300 nL per min using a lineargradient from 92 % A (0.1% formic acid) to 22 % B (0,1% formic acid in 100 acetonitrile) over 93 min followedby a linear gradient from 22 % B to 35 % B over 20 min at a flowrate of 300 nL per min.

Mass spectra were acquired in positive ion mode applying automatic data-dependent switch between one Orbitrap survey MS scan in the mass range of 400 to 1200 m/z followed by HCD fragmentation and Orbitrap detection of the 15 most intense ions observed in the MS scan. Target value in the Orbitrap for MS scan was 1,000,000 ions at a resolution of 70,000 at m/z 200. Fragmentation in the HCD cell was performed at normalized collision energy of 31 eV. Ion selection threshold was set to 25,000 counts and maximum injection time was 100 ms for MS scans and 300 ms for MSMS scans. Selected sequenced ions were dynamically excluded for 60 sec.

The obtained data from the 30 LC-MS runs were searched using VEMS (4) and MaxQuant (5). Each of the three biological replicas were analyzed by LC-MS by three technical replicas. A standard human proteome database from UniProt (3AUP000005640) with permutated protein sequences, where Arg and Lys were not permutated, were specified for the search. Trypsin cleavage allowing a maximum of 4 missed cleavages was used. Carbamidomethyl cysteine was included as fixed modification. Methionine oxidation, N-terminal protein acetylation, N-terminal NH_3 loss, STY phosphorylation, deamidation of asparagine and glutamine were included as variable modifications. Additionally, 5 ppm mass accuracy was specified for precursor ions and 0.01 m/z for fragment ions. The false discovery rate (FDR) for protein identification was set to 1% for peptide and protein identifications. No restriction was applied for minimal peptide length for VEMS search. Identified proteins were divided into evidence groups as defined by Matthiesen *et al.* (6).

Western blot validation

Western blot analysis was performed for SPRY4, in the two cell lines previously analyzed by proteomics. In brief, 2D transwell co-cultures were performed, as described above, using C3948 and T235 ATC cell lines ($2x10^5$ cells/well) and THP-1 cells ($6x10^5$ cells/insert). After 24 h of indirect co-culture, protein extraction was performed using RIPA buffer (#89900, ThermoFisher Scientific, Massachusetts, USA), supplemented with 1x Sigma protease and phosphatase inhibitor cocktail (#P8340, Sigma-Aldrich, Missouri, USA). Total protein quantification was performed using the Pierce™ BCA protein assay kit (Thermo Fisher Scientific, Illinois, USA), according to the manufacturer's protocol. Protein quantification was performed in a clear 96-well plate and the absorbance was measured at 595 nm on a Microplate Absorbance Reader (iMARK™; Bio-Rad Laboratories, California, USA). Protein extracts were prepared in 5x Laemmli Buffer and β-mercaptoethanol (Sigma-Aldrich, Missouri, USA), in a dilution of 1:20, and then denatured at 100 °C for 5

min. For western blotting, 30 μg of total protein extracts were loaded and run in a Multi-PROTEIN® Tetra System (Bio-Rad Laboratories, California, USA), using 1x Running Buffer (#161-0772, Bio-Rad Laboratories, California, USA). BenchMark™ pre-stained protein ladder (Life Technologies, California, USA) was used as a molecular marker. Protein transfer was performed using polyvinylidene difluoride (PVDF) membranes (#1704157, Bio-Rad Laboratories, California, USA), reservoir stacks (Bio-Rad Laboratories, California, USA), and 1x (v/v) Transfer Buffer (#1610734, Bio-Rad Laboratories, California, USA), and placed in a Trans-Blot® Turbo™ Transfer System (Bio-Rad Laboratories, California, USA). The PVDF membranes were incubated, overnight, with an anti-SPRY4 rabbit polyclonal antibody (AB_2458875, Invitrogen, California, USA). The secondary antibody incubation for SPRY4 was done using goat polyclonal anti-rabbit conjugated with horseradish peroxidase (HRP) (#31460, ThermoFisher Scientific, Massachusetts, USA). Alfa-tubulin was used as an endogenous control gene. Primary antibody incubation was done using the anti-α-tubulin mouse monoclonal antibody (clone B-5-1-2; #T5168, Sigma-Aldrich, Missouri, USA), and a secondary incubation using the goat polyclonal anti-mouse conjugated with HRP (#31430, ThermoFisher Scientific, Massachusetts, USA). Revelation was performed with a Clarity™ Western ECL Blotting Substrate (Bio-Rad Laboratories, California, USA), that enhances the chemiluminescence, and reveled in a Molecular imager® ChemiDoc™ XRS+ (Bio-Rad Laboratories, California, USA). Analysis of SPRY4 protein expression by western blot was performed in three independent assays.

To analyze the efficacy of *SPRY4* silencing at protein level, the same procedure was performed, although using 10 μg of protein extracts and the SPRY4 rabbit monoclonal antibody (clone EPR12127, #ab176337, Abcam, Cambridge, UK). Secondary antibody incubation for SPRY4 was done using goat polyclonal anti-rabbit conjugated with horseradish peroxidase (HRP) (#31460, ThermoFisher Scientific, Massachusetts, USA). Beta-actin was used as an endogenous control gene. Primary antibody incubation was done using the anti-β-actin mouse monoclonal antibody (clone AC-15, #A5441, Sigma-Aldrich, Missouri, USA) and secondary antibody incubation was done using the secondary antibody goat polyclonal anti-mouse conjugated with HRP (#31430, ThermoFisher Scientific, Massachusetts, USA).

Supplementary Figures

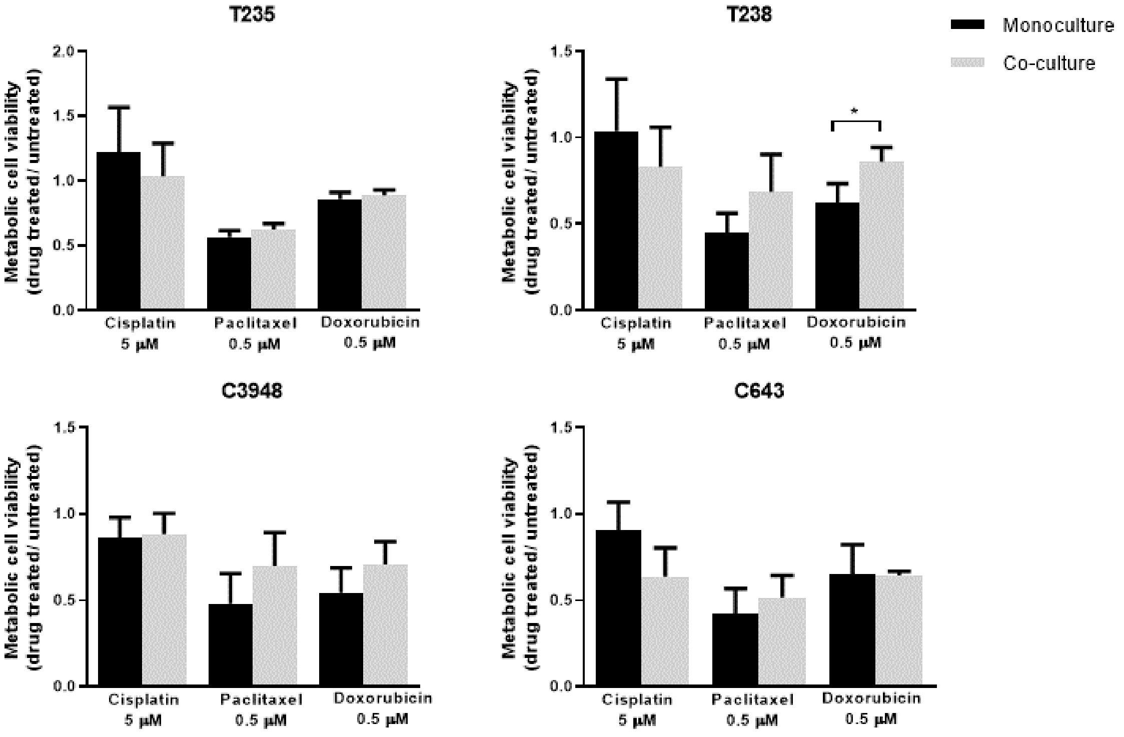

Figure S1. Drug resistance of ATC cells in the presence of macrophages. The metabolic viability of T235, T238, C3948 and C643 ATC cells, in monoculture or co-culture with THP-1-derived macrophages, after exposure to cytotoxic drugs (cisplatin, 5 µM; placlitaxel, 0.5 µM; doxorrubicin, 0.5 µM) for 1 h. Drug treated co- and monocultures were compared with the respective non-treated cultures. Treated/untreated ratios are presented for mono- (black) and co-cultures (gray) ($n = 3$). $^*p < 0.05$

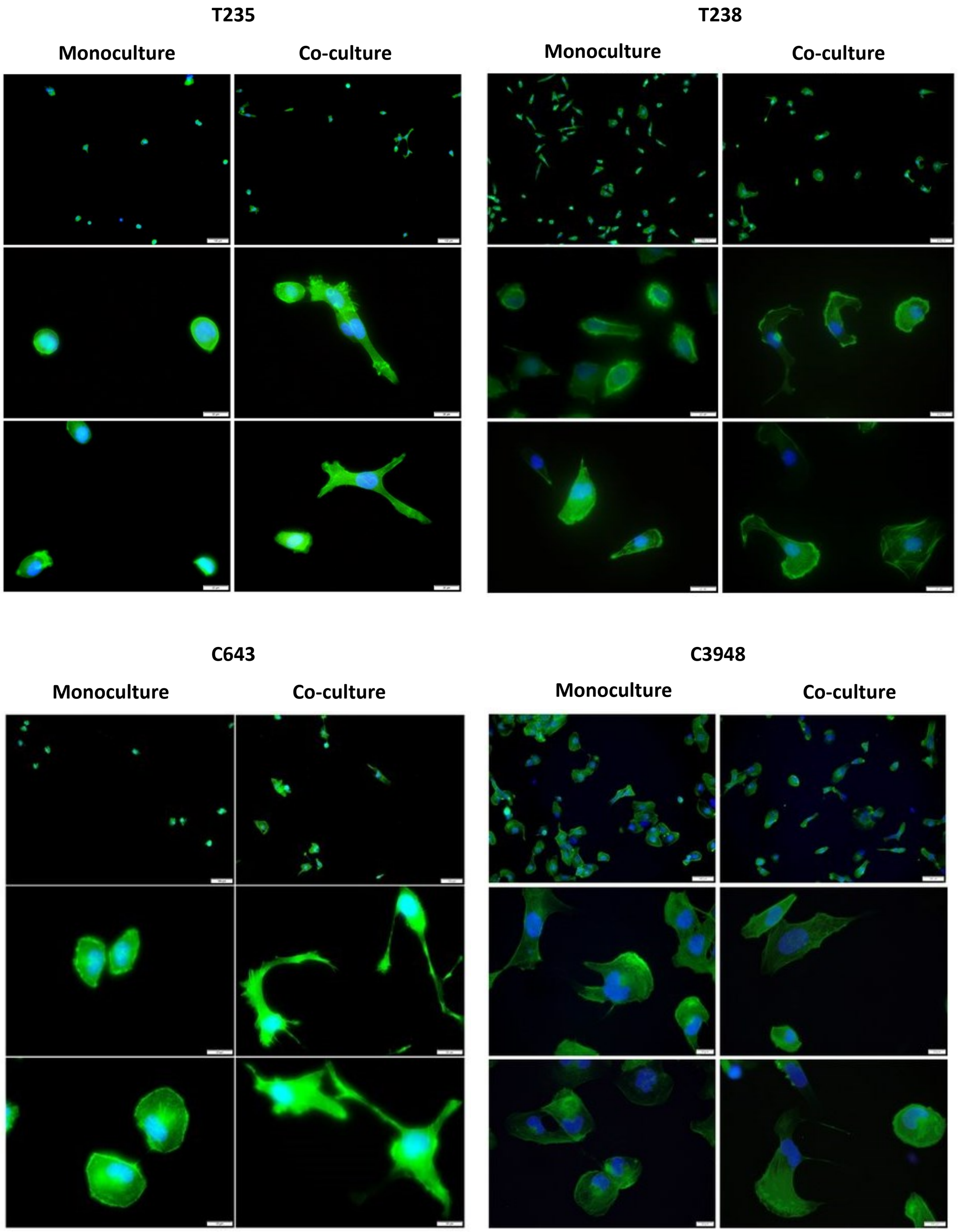

Figure S2. Actin cytoskeleton organization of ATC cells upon co-culture with THP-1-derived macrophages. Upon 24 h of co-culture, cancer cells were fixed, and F-actin (green) and the nucleus (blue) were stained. Representative photos ofactin cytoskeleton of T235 (n = 3), T238 (n = 1), C643 (n = 1) and C3948 (n = 3) ATC cells, either from mono or co-culturewith macrophages, are presented.

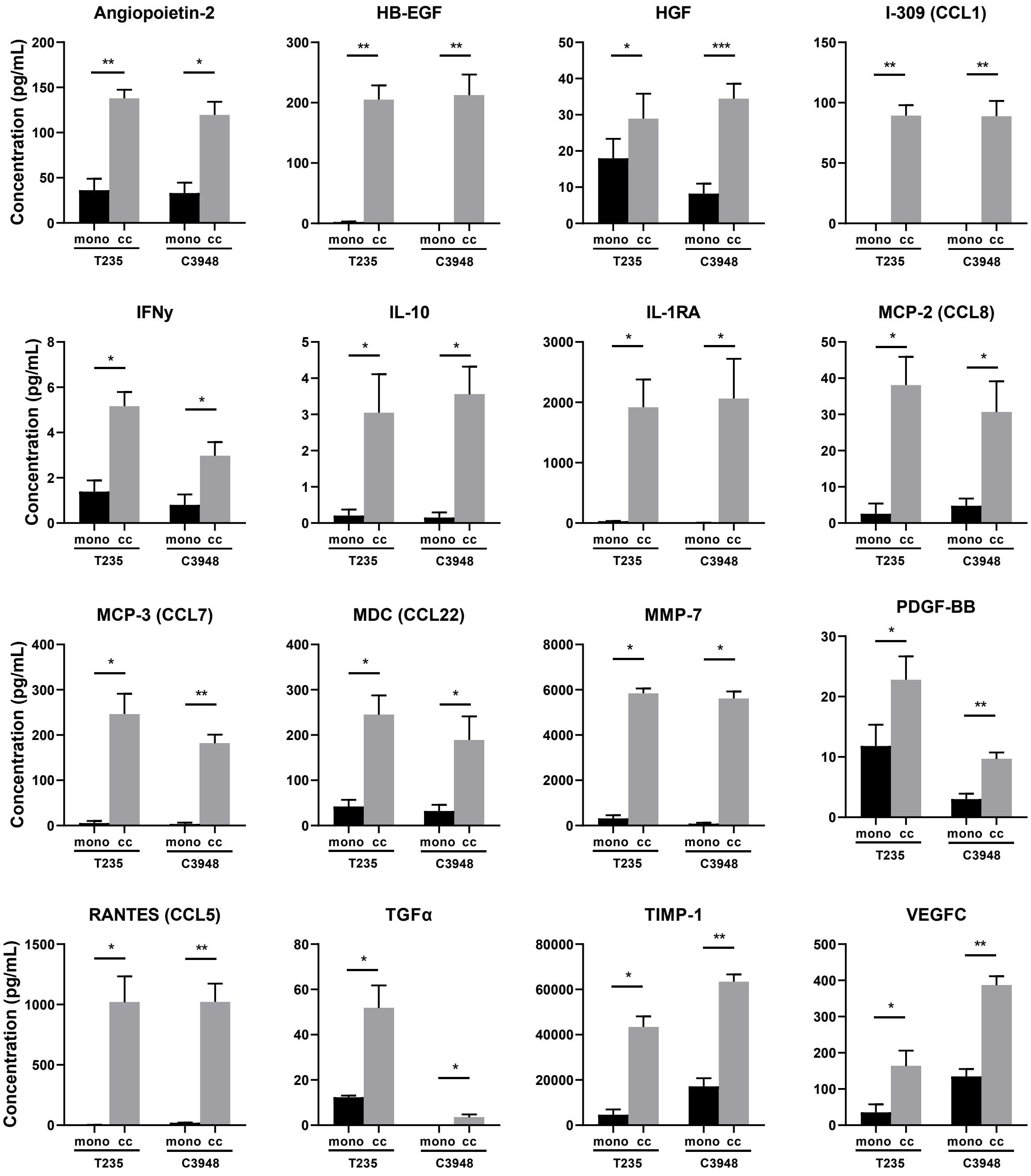

Figure S3. Secreted proteins significantly upregulated by the presence of macrophages in co-cultures with both ATC cell lines. The conditioned medium of T235/C3948-macrophage co-cultures and ATC monocultures was analyzed by multiplex array ($n = 3$). Co-cultures were compared with ATC monocultures. Multiple t-test analysis was performed. ns, non-significant, * $p < 0.05$, ** $p < 0.01$, *** $p < 0.001$.

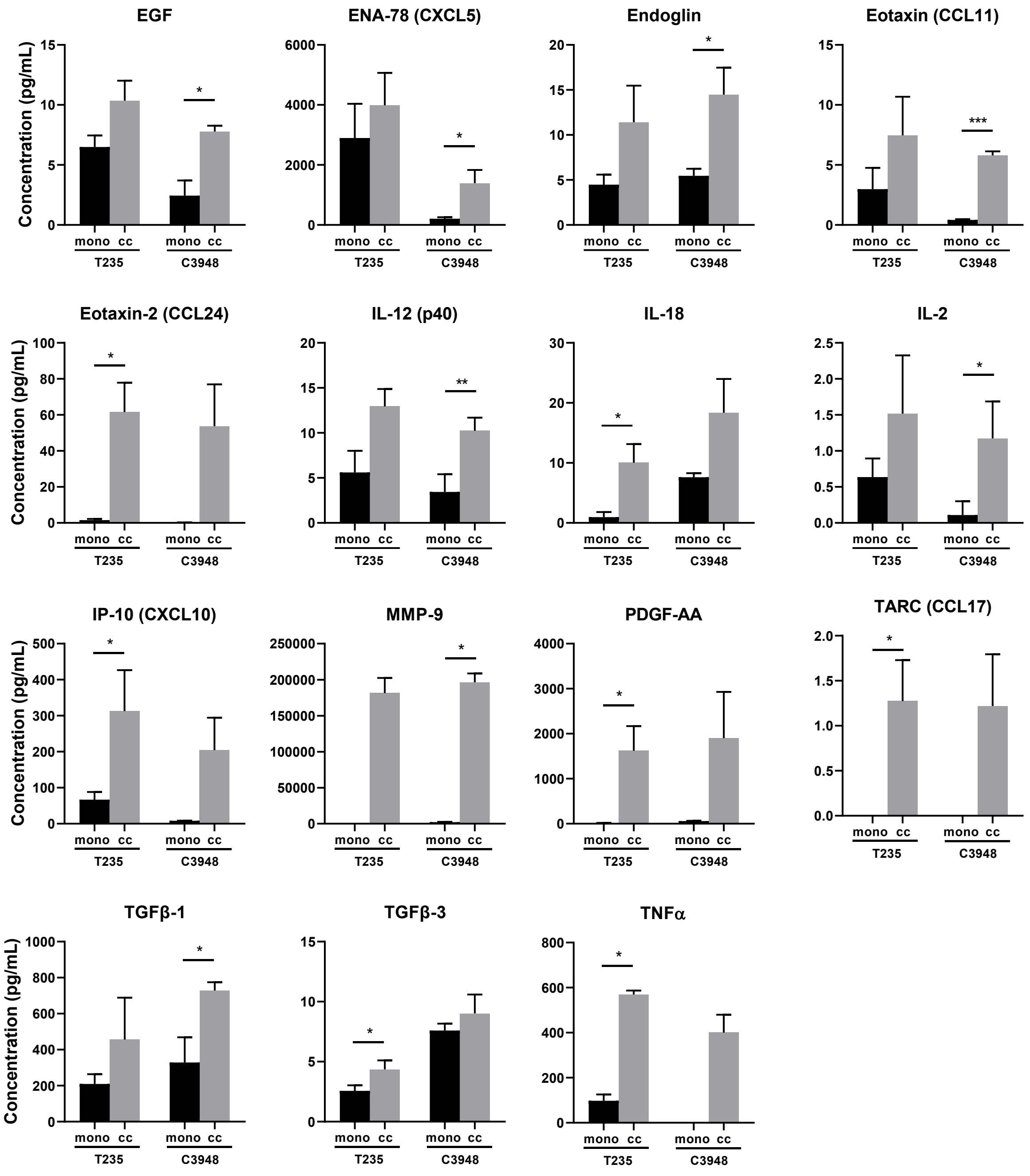

Figure S4. Secreted protein targets significantly upregulated in at least one of the cell lines co-cultures, but with the same tendency in both. The conditioned medium of T235/C3948-macrophage co-cultures and ATC monocultures was analyzed by multiplex array (n = 3). Co-cultures were compared with ATC monocultures. Multiple t-test analysis was performed. ns, non-significant, * $p < 0.05$, ** $p < 0.01$, *** $p < 0.001$.

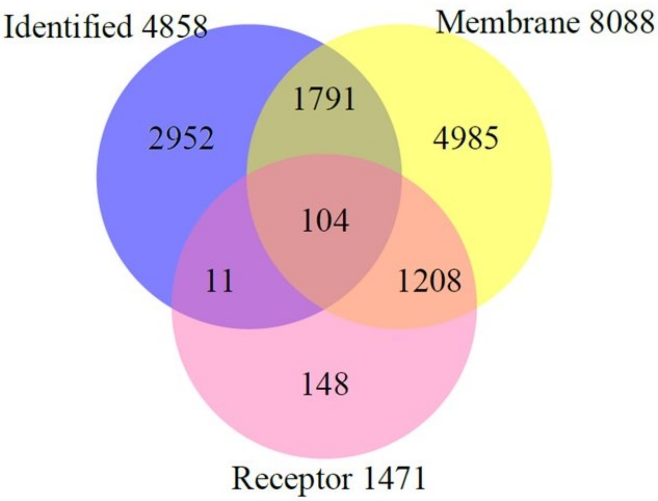

Figure S5. Venn diagram displaying overlap in identified proteins with UniProt annotated membrane proteins and receptors.

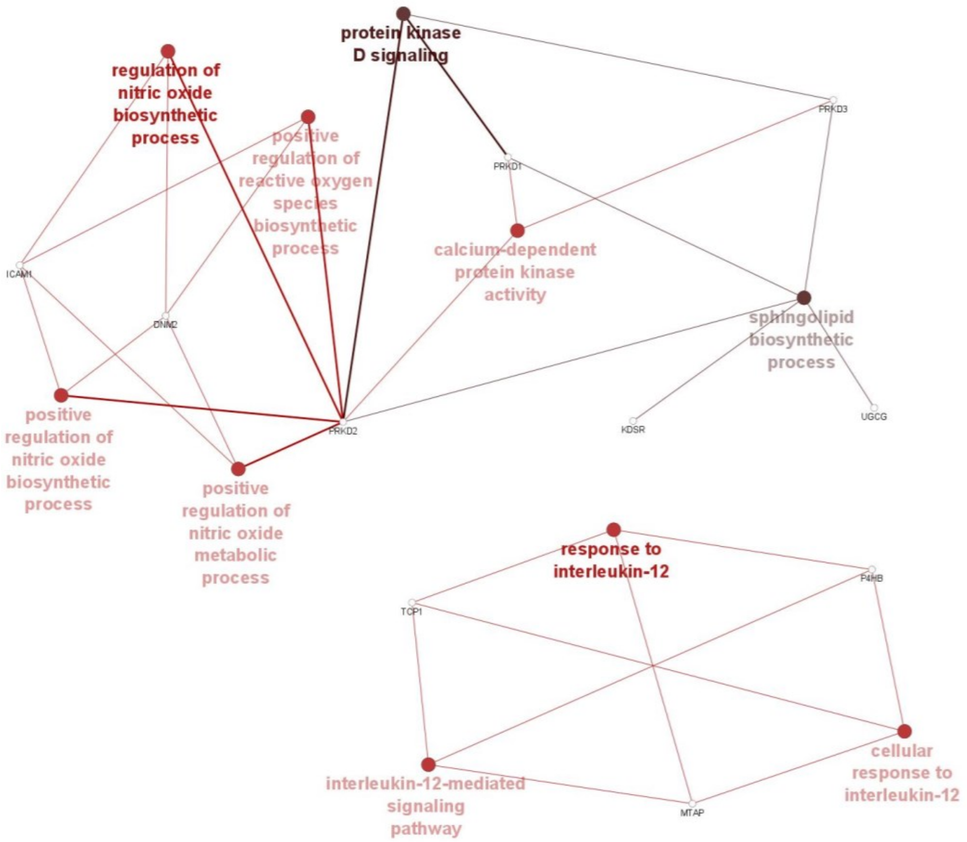

Figure S6. Proteomic analysis of C3948 ATC cells after co-culture with macrophages. Functional analysis of significantly altered targets in C3948 cells co-cultured with macrophages, when compared with monoculture. The presented protein-protein interaction network considers the biological processes significantly associated with the altered targets in C3948 cells co-cultured with macrophages (Cytoscape version 3.8, ClueGo/CluPedia apps). Darker nodes refer to the most significant associations.

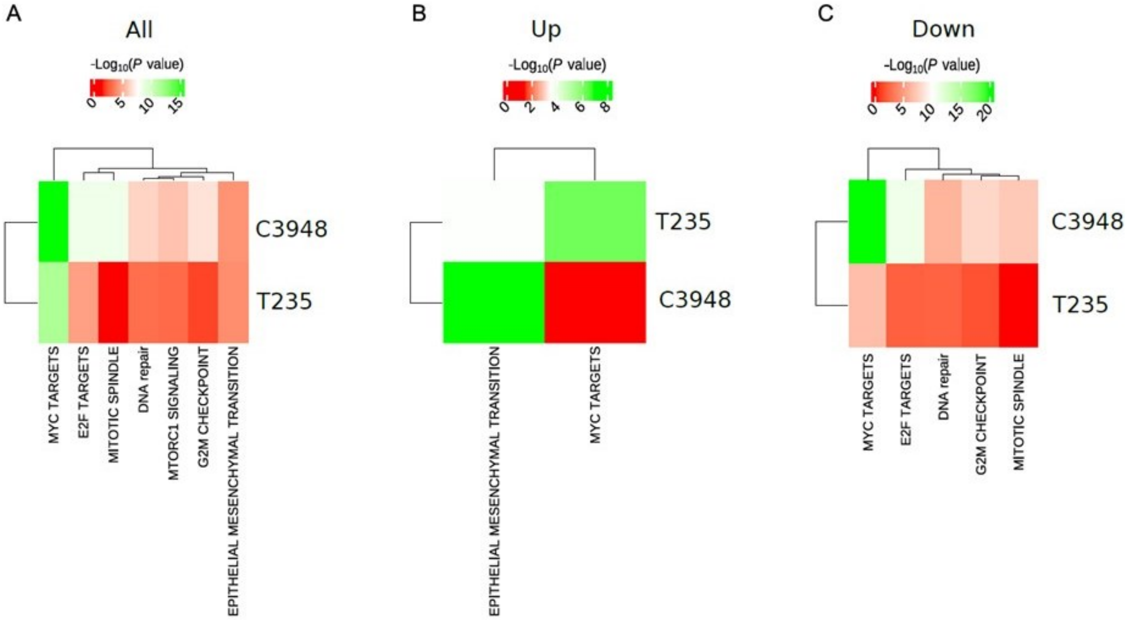

Figure S7. Heatmap displaying -log10 P enrichment significance of cancer hallmark proteins encoded as colors when submitting regulated proteins for C3948 and T235 (co-culture vs monoculture).

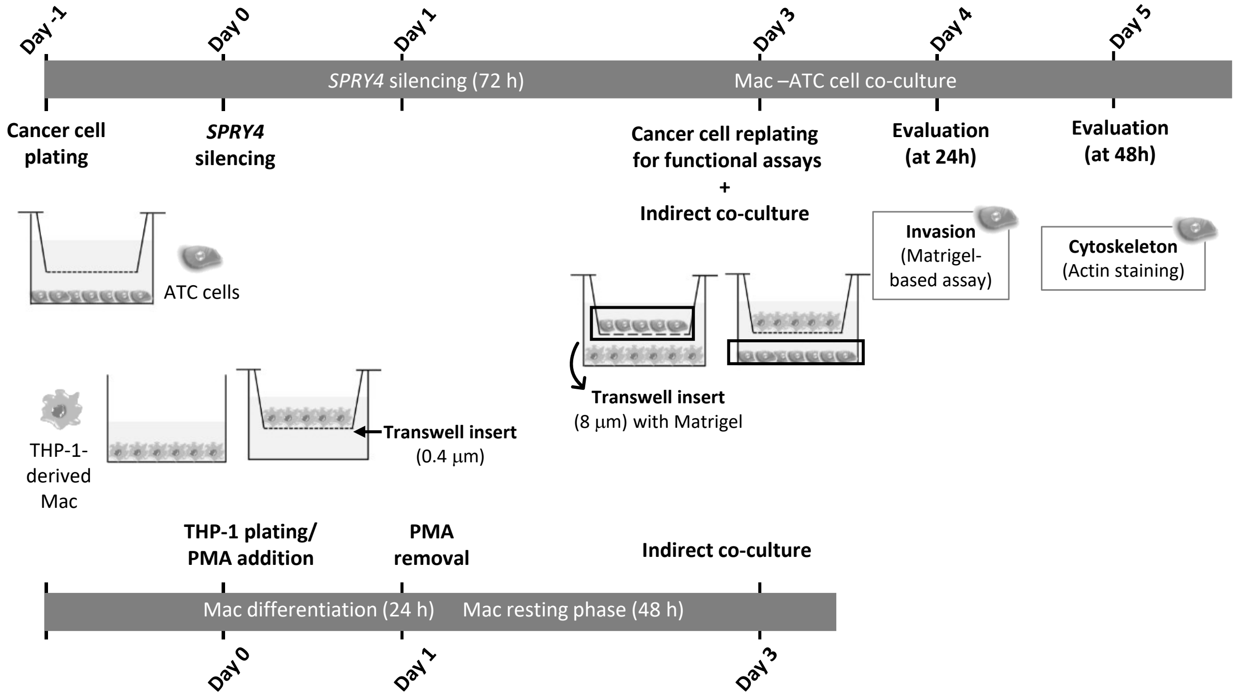

Figure S8. Schematic representation of the experimental procedure after *SPRY4* silencing. At day 0, *SPRY4* was silenced in ATC cells (T235 and C3948) for 72 h. In parallel, THP-1 monocytes were differentiated into macrophages for 24 h, in the presence of PMA, either in the bottom of the well or on the top of transwell inserts with pore size of 0.4 μm. At day 3, macrophage-ATC cell co-culture was performed by replating ATC cells indirectly with macrophages, either on the bottom compartment or on the top of transwell inserts with pore size of 8 μm coated with Matrigel. At 24 and 48 h after co-culture, ATC cell invasion and cytoskeleton were evaluated, respectively. ATC, anaplastic thyroid cancer; Mac, macrophages; PMA, phorbol-12-myristate-13-acetate.

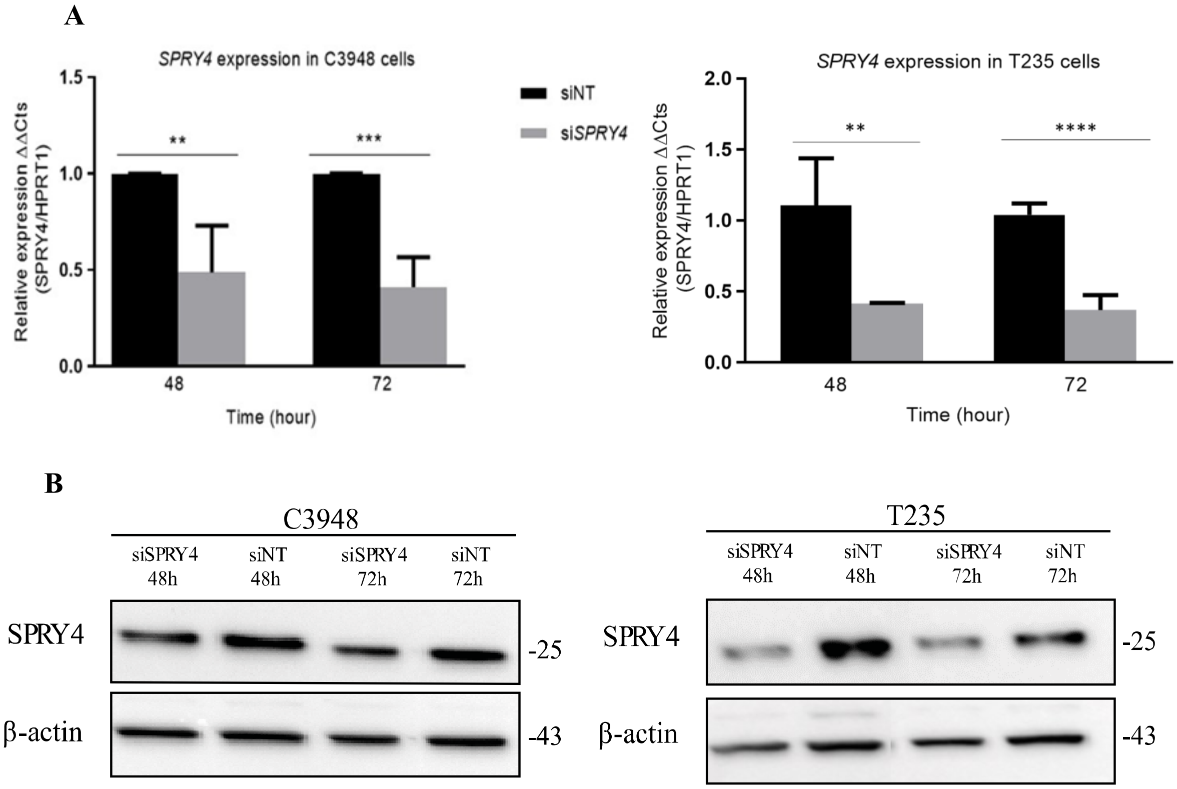

Figure S9. Efficient *SPRY4* silencing in ATC cell lines**.** (A) The silencing of *SPRY4* in C3948 cell line with 12.5 nM of siRNA resulted in an average reduction of 53% at 48h and 58% at 72 h in mRNA expression levels. The silencing of *SPRY4* in T235 cell line with 37.5 nM of siRNA resulted in an average reduction of 59% at 48h and 64% at 72 h in RNA expression levels (analyzed by qRT-PCR). As a transfection control, a non-targeting negative control siRNA (siNT) wasused. The bars represent the mean and standard deviation (SD). Data were analyzed using the parametric unpaired *t*-test ($n = 4$) ** $p < 0.01$, *** $p < 0.001$, **** $p < 0.0001$. (B) Western blot of representative assays with C3948 and T235 cells. SPRY4 was detected by a rabbit monoclonal anti-SPRY4 antibody and β-actin by a mouse monoclonal anti-β-actin antibody. SPRY4 is represented with a molecular weight of 25kDa, and b-actin with 43 kDa.

Supplementary References

1. Carvalho AS, Molina H, Matthiesen R. New insights into functional regulation in MS-based drug profiling. Sci Rep. 2016;6:18826.
2. Wiśniewski JR, Zougman A, Nagaraj N, Mann M. Universal sample preparation method for proteome analysis. Nat Methods. 2009;6(5):359-62.
3. El-Motiam A, Vidal S, de la Cruz-Herrera CF, Da Silva-Álvarez S, Baz-Martínez M, Seoane R, et al. Interplay between SUMOylation and NEDDylation regulates RPL11 localization and function. FASEB J. 2019;33(1):643-51.
4. Carvalho AS, Ribeiro H, Voabil P, Penque D, Jensen ON, Molina H, et al. Global mass spectrometry and transcriptomics array based drug profiling provides novel insight into glucosamine induced endoplasmic reticulum stress. Mol Cell Proteomics. 2014;13(12):3294-307.
5. Cox J, Mann M. MaxQuant enables high peptide identification rates, individualized p.p.b.-range mass accuracies and proteome-wide protein quantification. Nat Biotechnol. 2008;26(12):1367-72.
6. Matthiesen R, Pireto G, Amorim A, Aloria K, Fullaondo A, Carvalho AS, et al. SIR: Deterministic protein inference from peptides assigned to MS data. J Proteomics. 2012;75(13):4176-83.
